# A cross-sectional study of the role of epithelial cell injury in kidney transplant outcomes

**DOI:** 10.1172/jci.insight.188658

**Published:** 2025-04-15

**Authors:** Philip F. Halloran, Jessica Chang, Martina Mackova, Katelynn S. Madill-Thomsen, Enver Akalin, Tarek Alhamad, Sanjiv Anand, Miha Arnol, Rajendra Baliga, Mirosław Banasik, Christopher D. Blosser, Georg Böhmig, Daniel Brennan, Jonathan Bromberg, Klemens Budde, Andrzej Chamienia, Kevin Chow, Michał Ciszek, Declan de Freitas, Dominika Dęborska-Materkowska, Alicja Debska-Ślizień, Arjang Djamali, Leszek Domański, Magdalena Durlik, Gunilla Einecke, Farsad Eskandary, Richard Fatica, Iman Francis, Justyna Fryc, John Gill, Jagbir Gill, Maciej Glyda, Sita Gourishankar, Marta Gryczman, Gaurav Gupta, Petra Hruba, Peter Hughes, Arskarapurk Jittirat, Zeljka Jurekovic, Layla Kamal, Mahmoud Kamel, Sam Kant, Nika Kojc, Joanna Konopa, James Lan, Roslyn B. Mannon, Arthur Matas, Joanna Mazurkiewicz, Marius Miglinas, Thomas Mueller, Marek Myślak, Seth Narins, Beata Naumnik, Anita Patel, Agnieszka Perkowska-Ptasińska, Michael Picton, Grzegorz Piecha, Emilio Poggio, Silvie Rajnochová Bloudíčkova, Thomas Schachtner, Soroush Shojai, Majid L.N. Sikosana, Janka Slatinská, Katarzyna Smykal-Jankowiak, Ashish Solanki, Željka Veceric Haler, Ondrej Viklicky, Ksenija Vucur, Matthew R. Weir, Andrzej Wiecek, Zbigniew Włodarczyk, Harold Yang, Ziad Zaky, Patrick T. Gauthier, Christian Hinze

**Affiliations:** 1 Alberta Transplant Applied Genomics Centre and; 2 University of Alberta, Edmonton, Alberta, Canada.; 3 Montefiore Medical Center, Bronx, New York, USA.; 4 Washington University at St. Louis, St. Louis, Missouri, USA.; 5 Intermountain Transplant Services, Murray, Utah, USA.; 6 University of Ljubljana, Ljubljana, Slovenia.; 7 Tampa General Hospital, Tampa, Florida, USA.; 8 Medical University of Wrocław, Wrocław, Poland.; 9 University of Washington, Seattle, Washington, USA.; 10 Medical University of Vienna, Vienna, Austria.; 11 Johns Hopkins University School of Medicine, Baltimore, Maryland, USA.; 12 University of Maryland, Baltimore, Maryland, USA.; 13 Charité – Universitätsmedizin Berlin, Berlin, Germany.; 14 Medical University of Gdańsk, Gdańsk, Poland.; 15 The Royal Melbourne Hospital, Parkville, Victoria, Australia.; 16 Warsaw Medical University, Warsaw, Poland.; 17 Manchester Royal Infirmary, Manchester, United Kingdom.; 18 University of Wisconsin, Madison, Wisconsin, USA.; 19 Pomeranian Medical University, Szczecin, Poland.; 20 Hannover Medical School, Hannover, Germany.; 21 Cleveland Clinic Foundation, Cleveland, Ohio, USA.; 22 Henry Ford Transplant Institute, Detroit, Michigan, USA.; 23 Medical University of Białystok, Białystok, Poland.; 24 St. Paul’s Hospital, Vancouver, British Columbia, Canada.; 25 Wojewodzki Hospital, Poznan, Poland.; 26 Virginia Commonwealth University, Richmond, Virginia, USA.; 27 Institute for Experimental and Clinical Medicine, Prague, Czech Republic.; 28 University Hospital Cleveland Medical Center, Cleveland, Ohio, USA.; 29 University Hospital Merkur, Zagreb, Croatia.; 30 University of Alabama at Birmingham, Birmingham, Alabama, USA.; 31 University of Minnesota, Minneapolis, Minnesota, USA.; 32 Vilnius University Hospital Santaros Klinikos, Vilnius, Lithuania.; 33 University Hospital Zurich, Zurich, Switzerland.; 34 PinnacleHealth Transplant Associates, UPMC, Harrisburg, Pennsylvania, USA.; 35 Silesian Medical University, Katowice, Poland.; 36 University Hospital No. 1, Bydgoszcz, Poland.

**Keywords:** Nephrology, Transplantation, Molecular diagnosis, Organ transplantation

## Abstract

Background. Expression of acute kidney injury–associated (AKI-associated) transcripts in kidney transplants may reflect recent injury and accumulation of epithelial cells in “failed repair” states. We hypothesized that the phenomenon of failed repair could be associated with deterioration and failure in kidney transplants.

Methods. We defined injury-induced transcriptome states in 4,502 kidney transplant biopsies injury-induced gene sets and classifiers previously developed in transplants.

Results. In principal component analysis (PCA), PC1 correlated with both acute and chronic kidney injury and related inflammation and PC2 with time posttransplant. Positive PC3 was a dimension that correlated with epithelial remodeling pathways and anticorrelated with inflammation. Both PC1 and PC3 correlated with reduced survival, with PC1 effects strongly increasing over time whereas PC3 effects were independent of time. In this model, we studied the expression of 12 “new” gene sets annotated in single-nucleus RNA-sequencing studies of epithelial cells with failed repair in native kidneys. The new gene sets reflecting epithelial-mesenchymal transition correlated with injury PC1 and PC3, lower estimated glomerular filtration rate, higher donor age, and future failure as strongly as any gene sets previously derived in transplants and were independent of nephron segment of origin and graft rejection.

Conclusion. These results suggest 2 dimensions in the kidney transplant response to injury: PC1, AKI-induced changes, failed repair, and inflammation; and PC3, a response involving epithelial remodeling without inflammation. Increasing kidney age amplifies PC1 and PC3.

Trial registration. INTERCOMEX (ClinicalTrials.gov NCT01299168); Trifecta-Kidney (ClinicalTrials.gov NCT04239703).

Funding. Genome Canada; Natera, Inc.; and Thermo Fisher Scientific.

## Abstract 2

Genes expressed in epithelial cells with failed repair are associated with dysfunction and failure in kidney transplants, and are amplified by donor age.

## Introduction

The molecular changes induced in response to parenchymal injury are important in acute kidney injury (AKI) and chronic kidney disease (CKD) and are associated with reduced function and progression to failure (1, 2). AKI and CKD are difficult to study molecularly in native kidneys because these conditions are not uniformly biopsied. In contrast, kidney transplants with dysfunction are frequently biopsied and universally experience parenchymal injury from donation implantation, rejection, drug toxicity, and other stresses, making kidney transplant biopsies of interest for exploring the spectrum of the kidney response to injury. In previous bulk transcriptomics studies, we used genome-wide microarrays to identify transcript sets characteristic of AKI and CKD. AKI in mouse kidney isografts (3) and human kidney transplant biopsies (4) manifested increased expression of transcripts such as vimentin and versican (VCAN) and decreased expression of many transcripts related to normal kidney function and metabolism, i.e., dedifferentiation. CKD (or atrophy-fibrosis) in transplant biopsies was associated with a separate constellation of changes: increased expression of transcripts representing plasma cells, e.g., immunoglobulin transcripts (IGTs) (5); mast cell transcripts (MCATs), e.g., CPA3 and FCER1A (6); and others such as CXCL6, a chemokine increased in progressing CKD but not in AKI (6). We also developed injury-related classifiers based on low estimated glomerular filtration rate (eGFR) (lowGFR_Prob_) (7), proteinuria (Prot_Prob_) (8), and histologic atrophy (ct>1_Prob_) and fibrosis (ci>1_Prob_) (9). These CKD- and AKI-related transcript sets and classifier scores predicted progression to failure more strongly than did rejection-associated transcripts (7, 10), even in kidneys with rejection (1, 11), indicating that molecular injury states were a final common pathway to failure.

In these studies, we developed a model of kidney transplant injury in 1,526 transplant biopsies and used principal component analysis (PCA) and archetypal analysis (AA) to describe variance in injury-induced gene expression (2, 7, 12). PCA revealed PC1 (all injury) and PC2 (time) but also identified PC3, a previously unknown dimension of injury-related variance that correlated with the expression of genes related to epithelial structure, e.g., par-3 family cell polarity regulator (PARD3), involved in polarity and adherens junctions. AA revealed heterogeneity within AKI, distinguishing “AKI1” and “AKI2,” and within CKD, distinguishing “mild CKD” from “CKDAKI,” a CKD state with AKI changes.

The expression of AKI-associated transcripts in kidney transplants may reflect not only recent injury but also accumulation of epithelial cells in “failed repair” states. This concept arises from mRNA-sequencing studies of the single epithelial cell nuclei from native kidneys with AKI (13–18). Failed repair manifested as populations of injured tubule epithelial cells, believed to be arrested at the G2/M cycle phase and sometimes manifesting a senescence-associated secretory phenotype (13, 19). Hinze et al. (14) demonstrated 4 distinct, hierarchically interconnected “new” injury-induced cell states by transcriptome patterns: New1, oxidative stress; New2, hypoxia; New3, interferon response; and New4, epithelial-to-mesenchymal transition (EMT). Transcriptome differences between individuals were driven by the abundance of cells in these 4 injury states. Similar transcripts develop in human kidney transplants (16, 20), and at least in rats, such transcripts are increased by aging (21).

We hypothesized that this failed repair phenomenon could be associated with deterioration and failure in kidney transplants (14, 16). The present analysis aimed to expand the transplant injury model to increase power and to test the relevance of transcript sets reflecting the epithelial cell failed repair states in the new model. We hoped to define the associations of these injury and failed repair states with function and outcomes in kidney transplants, including their relationship to donor age, and the implications for the responses to injury in native kidneys.

## Results

### The biopsy population.

**Figure 1:**
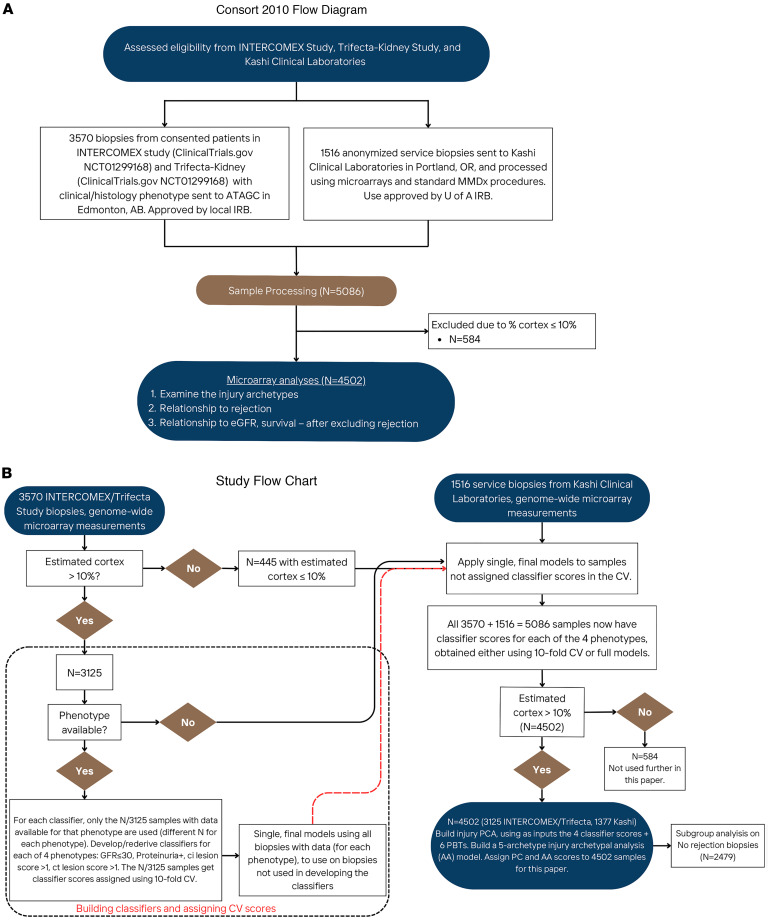
CONSORT diagram and study design. (**A**) CONSORT diagram showing biopsy inclusion for the INTERCOMEX and Trifecta-Kidney Study (*N* = 4,502) combined dataset. (**B**) Study design. ATAGC, Alberta Transplant Applied Genomics Centre; CV, cross-validation; MMDx, Molecular Microscope Diagnostic System.

We collected 4,502 biopsies (86% for indications) with a median time of biopsy posttransplant (TxBx) of 367 days (range 1 day to 45 years) (Figure 1 and Table 1). To ensure that the injury measurements were independent of molecular rejection measurements, the present study separately analyzes injury in the whole 4,502-biopsy population and in the subset of 2,479 biopsies automatically assigned to the “no rejection” archetype (22, 23). Note that because some no rejection archetype biopsies have subthreshold rejection activity (23), this group should be considered as minimal rejection. The demographics of the whole population and the no rejection subset were similar (Table 1).

### Developing a PCA/AA injury model in 4,502 biopsies.

**Figure 2:**
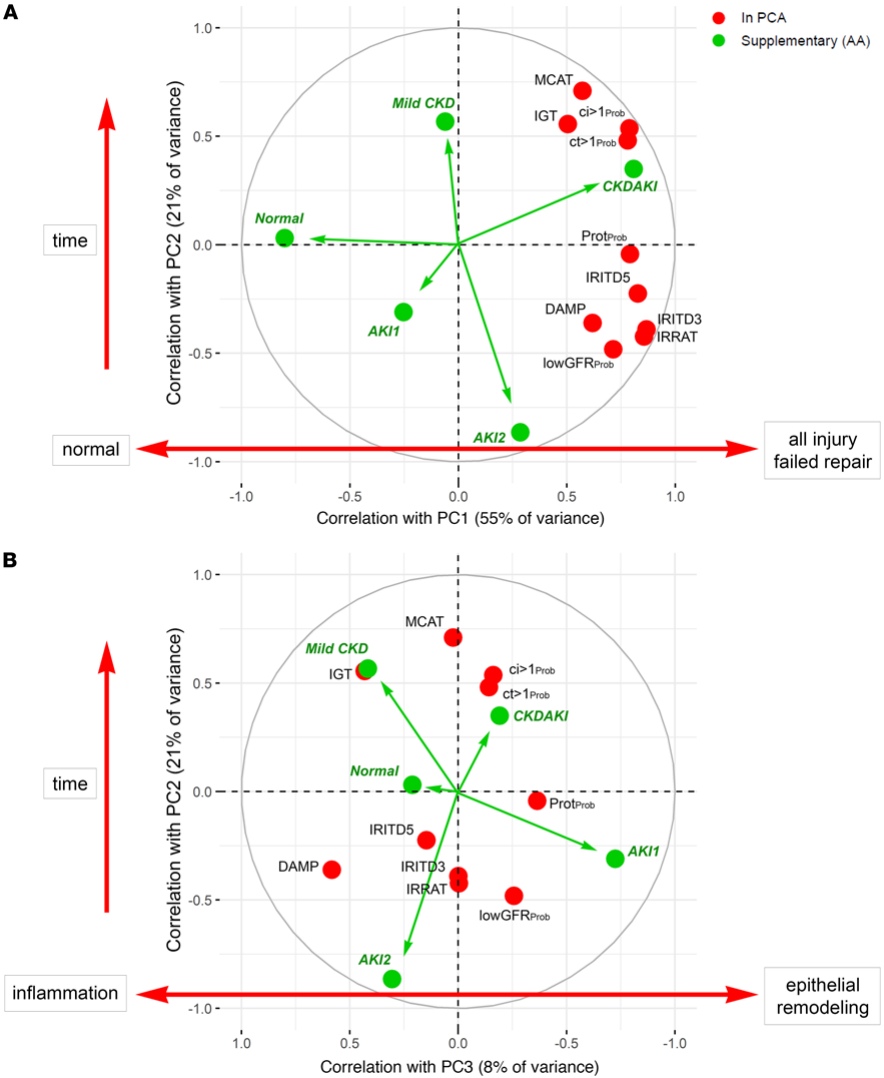
Factor maps in the *N* = 4,502 kidney transplant biopsy population showing the correlations between the input variables (red circles) and the principal components in the *N* = 4,502 kidney transplant biopsy population. AA groups are shown in green. The correlations between the PCA input variables and the PC scores are shown as factor maps in (**A**) PC2 vs. PC1 and (**B**) PC2 vs. PC3.

Molecular injury in each biopsy was assessed using 10 published injury-related multigene scores developed in transplants: 6 transcript sets (Supplemental Table 1; supplemental material available online with this article; https://doi.org/10.1172/jci.insight.188658DS1) and 4 classifiers (Supplemental Table 2). Unlike the earlier model (7), normal kidney transcripts were not included as inputs in the expanded model because we found that they did not improve the model. The correlations between the input scores and the resulting PCs are shown in Figure 2, A and B. Injury PC1 (55% of variance) was associated with increased scores for all 10 injury-related variables. Injury PC2 (21% of variance) was strongly related to TxBx, separating CKD-related scores (positive) from AKI-related scores (negative). PC3 (8% of variance) identified variance with AKI and within CKD.

AA identified 5 archetype clusters, labeled normal, AKI1, AKI2, mild CKD, and CKDAKI based on their injury-related features as described below. This was similar to the earlier model (2) but with 5 rather than 6 clusters to simplify the classification.

**Figure 3:**
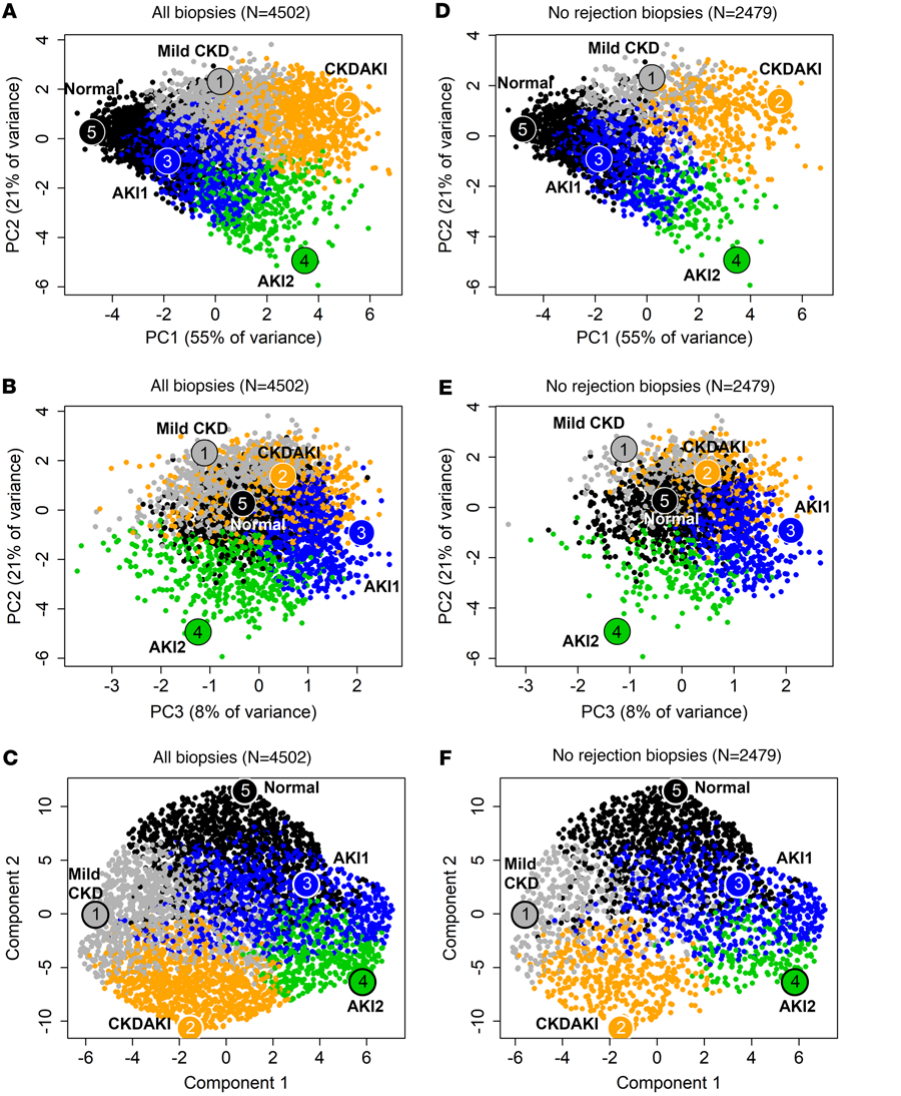
PCAs for biopsies. (**A**) PC2 vs. PC1 in all 4,502 biopsies, (**B**) PC2 vs. PC3 in all 4,502 biopsies, (**D**) PC2 vs. PC1 in 2,479 no rejection biopsies, (**E**) PC2 vs. PC3 in 2,479 no rejection biopsies, (**C**) UMAP visualization of the 4,502 population with variation compressed into 2 dimensions only, and (**F**) UMAP visualization of the 2,479 population of no rejection biopsies. PCAs and UMAP panels are colored by the 5-archetype injury model cluster assignments (normal, mild CKD, AKI1, AKI2, and CKDAKI). **D**–**F** show the no rejection samples within the original plots, rather than generating new PCAs/UMAPs using only the no rejection samples. MCAT, mast cell transcripts; IGT, immunoglobulin transcripts; ci>1_Prob_, ci lesion classifier; ct>1_Prob_, ct lesion classifier; Prot_Prob_, proteinuria classifier; IRITD5, injury and repair induced transcripts day 5; IRITD3, injury and repair induced transcripts day 3; IRRAT, injury and repair associated transcripts; DAMP, damage-associated molecular pattern transcripts; lowGFR_Prob_, probability of low GFR ≤ 30 cc/min/M^2^.

We distributed all 4,502 biopsies in PCA based on the 10 injury scores in Figure 3A, coloring biopsies by their injury archetype class. PC1 (55% of variance) strongly separated all injury groups from normal and separated AKI2 from AKI1 and CKDAKI from mild CKD. PC2 (21% of variance) separated all AKI (negative) from CKD (positive). Positive PC3 separated AKI1 from AKI2 and CKDAKI from mild CKD (Figure 3B).

Note that although archetypal clustering assigns the biopsies to groups, the actual distribution of biopsies is continuous. This interpretation is supported by using uniform manifold approximation and projection (UMAP), which collapses all injury-related variance into 2 dimensions, with no separation into distinct groups (Figure 3C).

In PCA or UMAP, the distribution of no rejection biopsies (Figure 3, D–F) was similar to all 4,502 biopsies (Figure 3, A–C).

### Relating molecular injury changes to the TxBx and eGFR.

**Figure 4:**
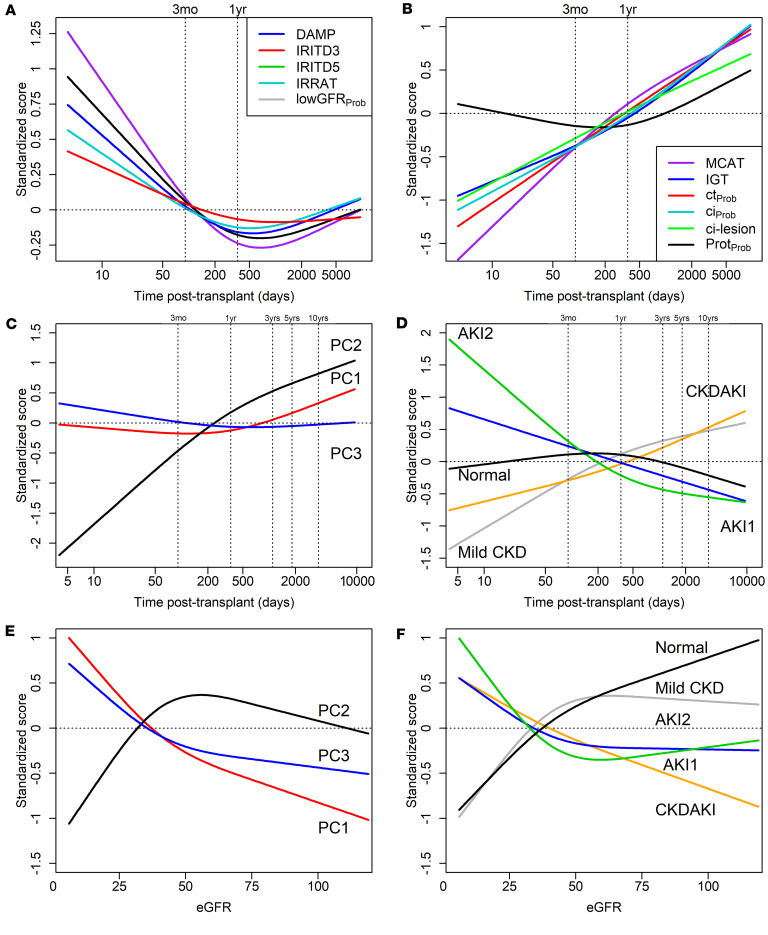
Relationships between injury principal component scores, injury archetypes, eGFR, and time posttransplant in all 4,502 biopsies with available data. (**A**) Restricted cubic splines showing the relationship between DAMP, IRITD3, IRITD5, IRRAT, and lowGFR_Prob_ and time posttransplant. (**B**) Restricted cubic splines showing the relationship between MCAT, IGT, ct>1_Prob_, ci>1_Prob_, ci lesion, and Prot_Prob_. (**C**) Restricted cubic splines showing the relationship between injury PC1, PC2, PC3 and time posttransplant in the *N* = 4,502 biopsy population. (**D**) Restricted cubic splines showing the relationship between injury archetypes and time posttransplant. Scores were standardized before analysis so that they could be shown on the same scale. (**E**) Restricted cubic splines showing the relationship between injury PC1, PC2, PC3, and eGFR. (**F**) Restricted cubic splines showing the relationship between injury archetypes and eGFR.

The 10 injury gene set (pathogenesis-based transcript sets, PBTs) scores used as input (*y* axis) are plotted as splines in Figure 4, A and B, and are strongly related to TxBx (*x* axis) as expected. The 5 AKI-related scores were highest early, fell to a trough at 1–2 years, and rose late (Figure 4A). The 4 CKD-related scores (MCAT, IGT, ci>1_Prob_, and ct>1_Prob_ rose steadily with time, as did the histologic fibrosis (ci) score; the proteinuria (Prot_Prob_) classifier score was high early and rose again late (Figure 4B).

The injury PC scores (Figure 4C) and archetype scores (Figure 4D) also correlated with TxBx (*x* axis). In Figure 4C, the relationship of injury PC1 score to time was U shaped: slightly higher early, lowest around 1 year, and rising late. Injury PC2 was strongly negative early and rose steadily with time. PC3 was higher at early times posttransplant but not strongly correlated with TxBx. In Figure 4D, the archetype scores (*y* axis) varied with TxBx. The normal score showed an inverted U shape with TxBx, the inverse of PC1: low early, peaking at about 1 year, and falling late. AKI1 and AKI2 archetype scores were high early (AKI2 > AKI1), decreased with TxBx, and never rose again (because late biopsies with AKI were in the CKDAKI cluster). Mild CKD and CKDAKI scores were low early and rose steadily with time.

The relationship to eGFR (*x* axis) is shown in Figure 4E for PC scores and Figure 4F for archetype scores. In Figure 4E, high injury PC1 and injury PC3 scores and low PC2 scores correlated with low eGFR. In Figure 4F, the eGFR also strongly correlated with the injury archetype scores; lowest with high AKI1, AKI2, and CKDAKI scores; and highest with high normal and mild CKD scores.

The TxBx and eGFR relationships in 2,479 no rejection biopsies were similar to those in all 4,502 biopsies (Supplemental Figure 1, A–D). The TxBx showed considerable overlap among the archetype groups (Supplemental Figure 1E).

### Relating injury PC and AA scores to donor age.

Donor age at transplantation correlated (ρ = correlation coefficient) with the PC scores: positively for PC1 (ρ = 0.10, *P* = 1 × 10^–5^); negatively for PC2 (ρ = –0.10, *P* = 2 × 10^–5^); and most strongly positively for PC3 (ρ = 0.20, *P* < 2 × 10^–16^) (Supplemental Table 3).

Donor age tended to be higher in AKI1 and CKDAKI, the groups with highest PC3 (Table 2). In all biopsies, compared with normal, mean donor age was older and percentage of donors aged ≥50 was higher in CKDAKI and AKI1 and lower in mild CKD. In early biopsies (≤42 days), AKI1 had older donor age than normal or AKI2 biopsies. In late biopsies, mean donor age and the percentage of donors aged ≥50 were higher in CKDAKI than normal.

### Top genes and Gene Ontology terms correlated with injury PC scores.

In Table 3, the top 10 transcripts correlating positively with injury PC1 were typical AKI-induced genes, potentially reflecting both recent injury and failed repair, e.g., VCAN. However, PC1 also correlated with some CKD features: For example, CXCL6 was highly correlated with PC1 (ρ = 0.75). We previously showed that CXCL6 correlates with CKD and is not AKI induced (24). VCAM1, a major feature of proximal convoluted tubule (PCT) failed repair cells in AKI (17, 18), also correlated strongly with PC1 (ρ = 0.70). The negative correlations with injury PC1 were related to decreased expression of genes related to metabolism or transport, e.g., KCl cotransporter SLC12A6. Injury PC2 negatively correlated genes were annotated as induced in early AKI, e.g., OLFM4 (4), whereas positively correlated genes were features of CKD, e.g., immunoglobulin and mast cell genes. Injury PC3 correlated positively with the expression of genes related to epithelial structure, e.g., PARD3, and negatively with genes expressed in inflammatory cells, e.g., LSP1. The results were similar in no rejection biopsies (Supplemental Table 4).

The Gene Ontology (GO) terms associated with the top 200 genes correlating positively or negatively with PC scores are shown in Tables 4, 5, and 6 (for all 4,502), and Supplemental Table 5 (for no rejection): PC1 positive, wound healing and inflammation; PC1 negative, metabolism and transport; PC2 positive, immunity (immunoglobulin genes); PC2 negative, mitosis; PC3 positive, epithelial organization; PC3 negative, inflammation and immunity.

### Top genes and GO terms correlated with injury archetype scores.

As shown in Supplemental Table 12 (all biopsies) and Supplemental Table 6 (no rejection biopsies), correlates with the normal score were reciprocal to those of PC1. The AKI1 score correlated positively with epithelial structure-related genes (e.g., PARD3) and negatively with plasma cell genes (e.g., CD79A and immunoglobulin genes), whereas the AKI2 score correlated positively with injury-induced genes, such as S100A8, and negatively with genes expressed in normal kidneys. The mild CKD score correlated positively with immunoglobulin genes and negatively with injury-inducible and mitosis inhibitor gene CDKN1A. The CKDAKI score correlated positively with genes induced in CKD (e.g., CXCL6) or both CKD and AKI (e.g., MMP7) and negatively with genes associated with normal kidney function (e.g., SLA12A6).

### Relating mitosis gene MKI67 with injury PC and AA scores.

Since AKI triggers mitosis, we studied expression of the MKI67 gene mitosis-associated gene (probe set 11721143_a_at). MKI67 was increased in TCMR, so we studied its relationships to injury in no rejection biopsies. MKI67 was highest in the early biopsies and correlated strongly positively with injury PC1 (ρ = 0.24; *P* = 6.8 × 10^–34^), negatively with injury PC2 (ρ = –0.30; *P* = 7.2 × 10^–52^), and weakly positively with injury PC3 (ρ = 0.07; *P* = 0.0008). MKI67 correlated negatively with the normal score (ρ = –0.29; *P* = 2.9 × 10^–49^) and most positively with the AKI2 score (ρ = 0.32; *P* = 1.1 × 10^–61^), weakly positively with the AKI1 score (ρ = 0.12; *P* = 5.8 × 10^–9^) and negatively with the mild CKD score (ρ = –0.18; *P* = 3.5 × 10^–19^), and did not correlate with the CKDAKI score (ρ = 0.003; *P* = 0.87).

### Conventional and molecular features of the archetype groups.

The timing, eGFR, molecular injury scores, and histologic fibrosis scores for each archetype group are summarized for all 4,502 biopsies in Table 7. AKI1 and AKI2 biopsies were both early, with low eGFR. AKI1 had lower macrophage and injury-induced molecular scores, less dedifferentiation (loss of kidney transcripts), and very low expression of immunoglobulin transcripts. CKDAKI and mild CKD were both late posttransplant, but CKDAKI had more fibrosis; more AKI-induced, mast cell, and macrophage transcripts; and lower eGFR, whereas mild CKD had more immunoglobulin transcripts. The features of the archetype groups were similar in the no rejection biopsies (Supplemental Table 7).

### Relationships of model features to survival after biopsy.

**Figure 5:**
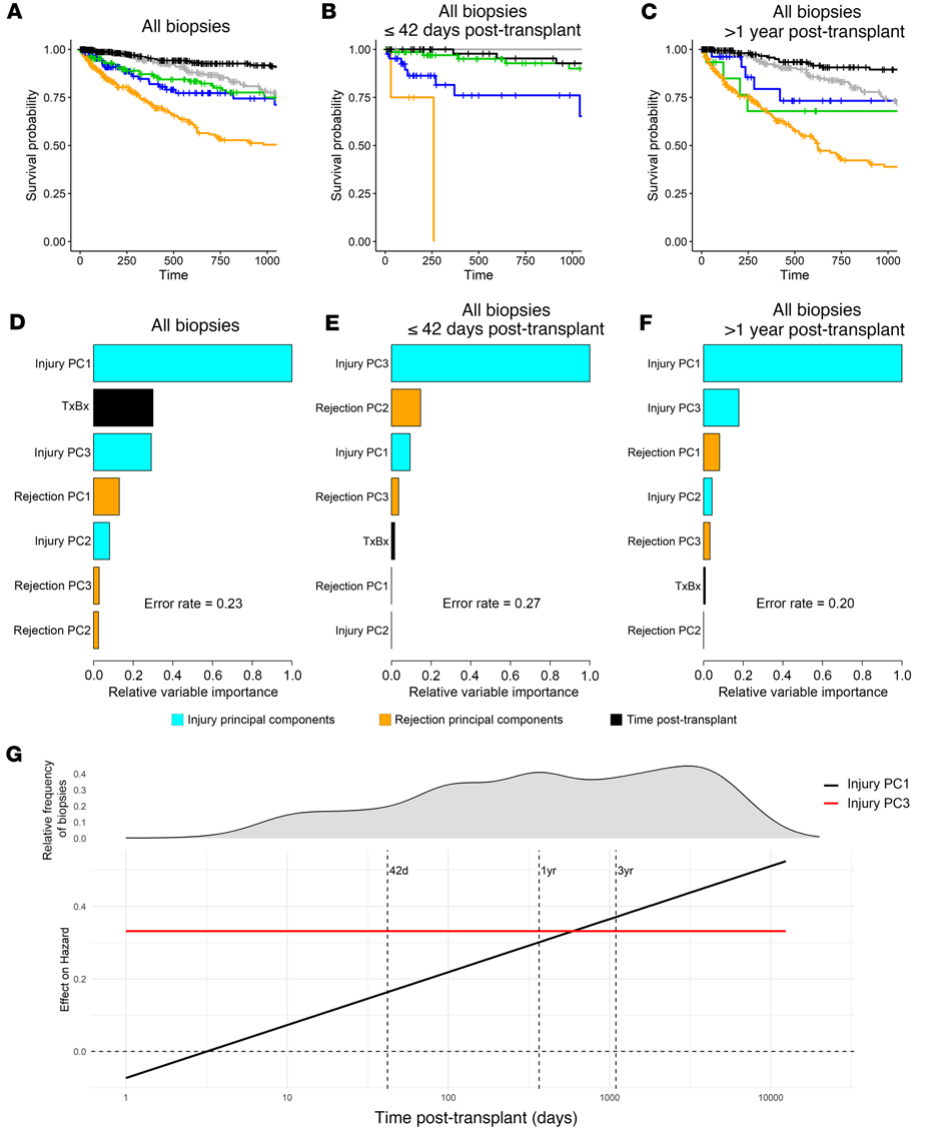
Visualizing the relationship between injury and 3-year postbiopsy death-censored survival (1 random biopsy per kidney) in all 4,502 biopsies. (**A**) Kaplan-Meier plots for the injury archetype groups in all 4,502 biopsies. (**B**) Kaplan-Meier plots for the injury archetype groups in biopsies ≤42 days. (**C**) Kaplan-Meier plots for the injury archetype groups in biopsies >1 year. (**D**–**F**) Relative variable importance plots from random survival forest analyses using injury PC1, PC2, and PC3; rejection PC1, PC2, and PC3; and time of biopsy posttransplant (TxBx) as predictors (**D**) in all 4,502 biopsies, (**E**) in biopsies ≤42 days posttransplant, (**F**) and in biopsies >1 year posttransplant. (**G**) Time-varying effects of injury PC1 and PC3 on the hazard of graft loss, estimated from a Cox proportional hazards model with interaction terms between time posttransplant and the injury PCs.

Figure 5A shows the actuarial 3-year survival for each archetype group (death censored, 1 random biopsy per kidney) in all cases with recorded follow-up. Postbiopsy 3-year graft survival was above 88% after normal biopsies; approximately 75% after AKI1, AKI2, or mild CKD biopsies; and less than 50% after CKDAKI biopsies.

After early biopsies (Figure 5B), survival was lowest after AKI1 biopsies. (There were too few early biopsies with CKDAKI or mild CKD to analyze reliably.)

Graft survival after late biopsies (Figure 5C) was similar to that in all biopsies, with the lowest survival in CKDAKI.

In random survival forest models comparing injury scores with rejection scores and TxBx, the best predictor of graft survival after biopsy in all biopsies (Figure 5D) was injury PC1 followed by injury PC3. In early biopsies (Figure 5E), the only strong predictor was injury PC3. PC1 was also the best predictor of graft survival in late biopsies (Figure 5F). In these multivariable analyses, rejection activity had little impact on survival once injury was considered, though some injury is caused by rejection.

Time of biopsy was a factor among all biopsies, with late biopsies having higher failure rates. The relative impact of PC1 and PC3 on hazard differed by the TxBx. PC1 had little relative impact on survival after early biopsies but steadily increased as the time of biopsy increased. PC3 had a constant negative relative impact, early and late (Figure 5G).

The results in no rejection biopsies were similar to the results in all biopsies (Supplemental Figure 2).

### Expression in transplants of gene sets representing epithelial cell failed repair states.

We used the new transplant injury model to explore in transplant biopsies the expression of the new epithelial cell injury gene sets identified in single-nucleus transcriptome studies of native kidneys with AKI (Supplemental Table 1) (14, 16). As stated earlier, those studies found cells from various nephron segments to be in 1 of 4 distinct biological states: oxidative stress (New1), hypoxia (New2), interferon response (New3), and EMT (New4). For transplant studies, we selected new injury gene sets derived from single nuclei from cells localized in the proximal tubule (PT), thick ascending limb (TAL), and distal convoluted tubule (DCT). However, the TAL-New1 gene set had only 1 probe set, so we substituted the thin limb (tL)-New1 to have each biological state represented by 3 different segments. (We stress that the derivation of genes in various cell types does not imply that their expression in bulk transcriptomic data is necessarily attributable to that cell type.)

**Figure 6:**
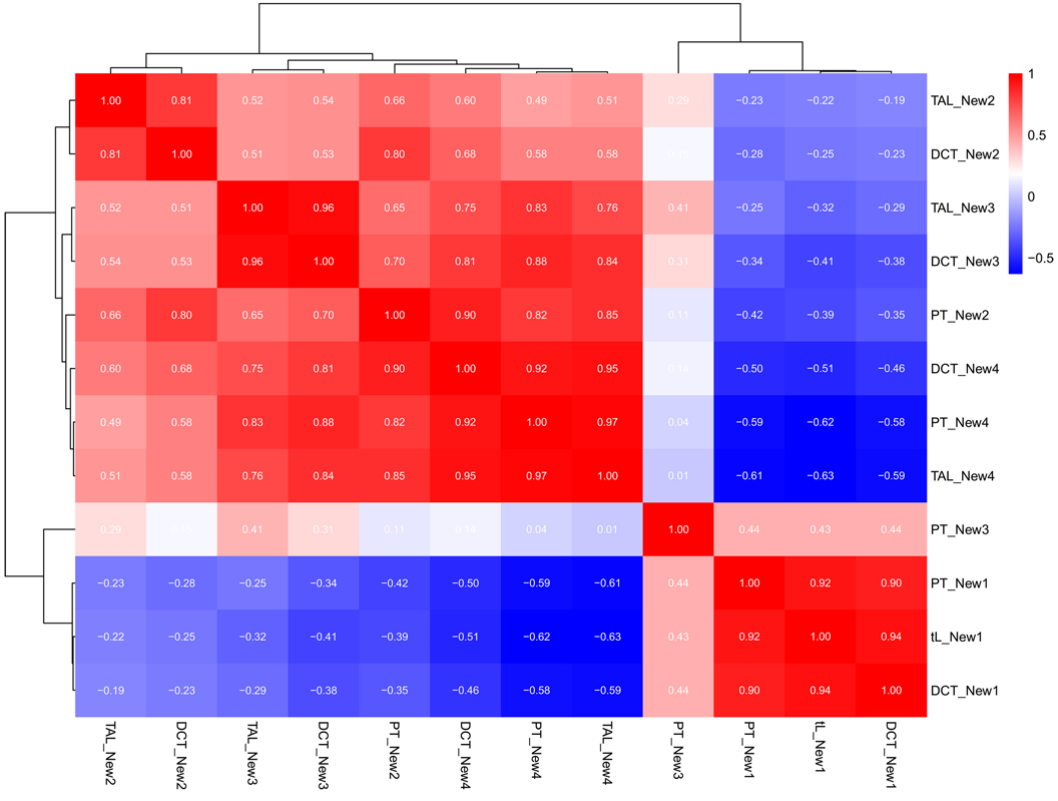
Heatmap showing pairwise Spearman correlations between the PBT scores of the 12 new gene sets in the N = 4,502 data set.

We studied the mean expression of the 12 gene sets (14) in the transplant model. Having established that injury changes were similar in all biopsies and no rejection biopsies, we used the full 4,502-transplant population. The heatmap in Figure 6 demonstrates the relationship between the mean expression for each set and that of the other 11 sets in the transplant biopsies. The gene set mean scores correlated strongly with the others in their biological state but not with their nephron segment of origin. (PT_New3 emerged as an outlier and is not discussed further.) The New4 sets strongly correlated with the New2 and New3 groups and anticorrelated with the New1 sets. Thus, the new gene sets annotated as biological states in native kidneys behaved as coherent sets in the transplant biopsies.

**Figure 7:**
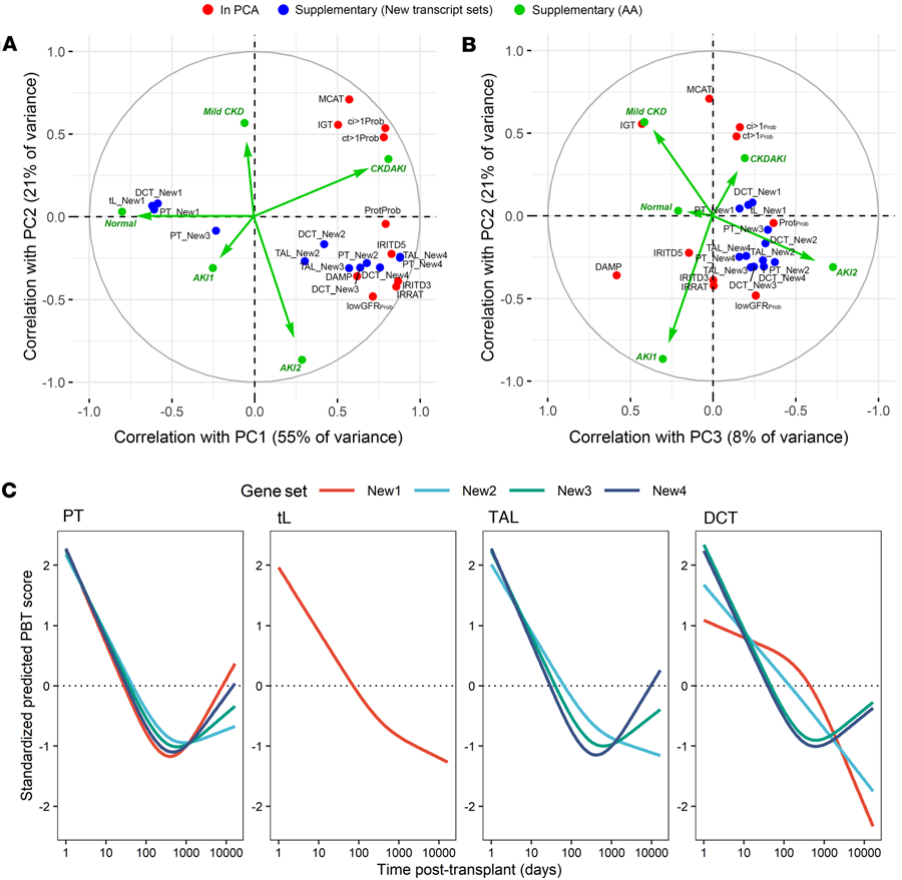
Factor maps in the *N* = 4,502 kidney transplant biopsy population showing the correlations between the input variables (red circles) and the principal components. Additional variables of interest (injury archetype scores in green and gene sets from ref. 14 as blue circles) were not used for the analysis but were projected as supplementary variables into the original PCA in (**A**) PC2 vs. PC1 and (**B**) PC2 vs. PC3. (**C**) Restricted cubic splines showing the relationship between the PBT scores for the 12 new gene sets and log(time posttransplant). *N* = 4,301 samples with TxBx available. MCAT, mast cell transcripts; IGT, immunoglobulin transcripts; ci>1_Prob_, ci lesion classifier; ct>1_Prob_, ct lesion classifier; Prot_Prob_, proteinuria classifier; IRITD5, injury and repair induced transcripts day 5; IRITD3, injury and repair induced transcripts day 3; IRRAT, injury and repair associated transcripts; DAMP, damage-associated molecular pattern transcripts; GFR, probability of low GFR ≤ 30 cc/min/M^2^; New1, oxidative stress; New2, hypoxia; New3, inflammation; New4, EMT; PT, proximal tubule; TAL, thick ascending limb; tL, thin limb; DCT, distal convoluted tubule.

To see the relationship of the new gene sets to the PC scores, Figure 7 projects the 12 new scores (blue symbols) onto the factor map for the transplant injury model presented in Figure 2. The 10 transplant-derived input scores are red symbols; the 5 injury archetype scores are green arrows and symbols; and the new gene set means are blue symbols. In Figure 7A, The New2, New3, and New4 scores correlated positively with PC1, the strongest being the New4 groups representing EMT. They correlated positively with AKI-related gene sets defined in transplants (IRRAT, IRITD3, IRITD5, and DAMP) and injury classifier lowGFR_Prob_. All correlated negatively with PC2. In contrast, the New1 gene sets correlated negatively with PC1, similar to the normal archetype score, with no association with PC2.

All 12 new gene sets, even the New1 sets, correlated positively with PC3 (Figure 7B).

The correlation coefficients of the new gene sets with PCA and archetype scores are shown in Supplemental Table 8.

Thus, new gene sets representing epithelial cell states derived in single epithelial cell nuclei from native kidneys with AKI strongly correlated with the injury dimensions in 4,502 kidney transplant biopsies, the strongest being the New4 EMT-related sets. The nephron segmental location of the single cells was not important.

The time courses of the new gene set scores in the 4,502 transplant biopsies indicated that all standardized scores were the highest initially and decreased in the first year posttransplant (Figure 7C). However, the New3 and New4 gene sets universally increased after 1 year posttransplant, similar to the AKI-related scores in Figure 4A. The New1 and New2 gene sets consistently decreased over time, except for in PT, which showed an increase after 1 year.

### Association of mean new injury scores with graft survival, eGFR, and donor age.

Table 8 presents the associations of each new injury gene set with 3-year death-censored graft survival (Cox analysis), with eGFR, and with donor age in the 4,502 kidney transplant biopsies. We included some injury gene sets derived from kidney transplants for comparison: IRRAT30, IRITD3, and IRITD5. In Table 8, the gene sets are ordered by their *P* value in the survival analysis. The injury PC scores are shown separately.

The 3 New4 gene set scores were the strongest predictors of graft survival, slightly better than the IRRAT gene set derived in transplant biopsies; injury PC1 was the strongest predictor. The New1 gene sets were protective, i.e., associated with decreased risk.

The New4 injury gene sets were the strongest correlates of depressed eGFR, stronger than the injury scores derived in kidney transplants, even injury PC1. The New1 gene sets correlated with higher eGFR.

The New4 gene sets all correlated positively with donor age. The New1 gene sets did not correlate with age. In this analysis, PC3 had the strongest correlation with donor age, as in our earlier analysis (7).

Similar results were found when the same analyses were done in the 2,479 no rejection biopsies (Supplemental Table 9).

**Figure 8:**
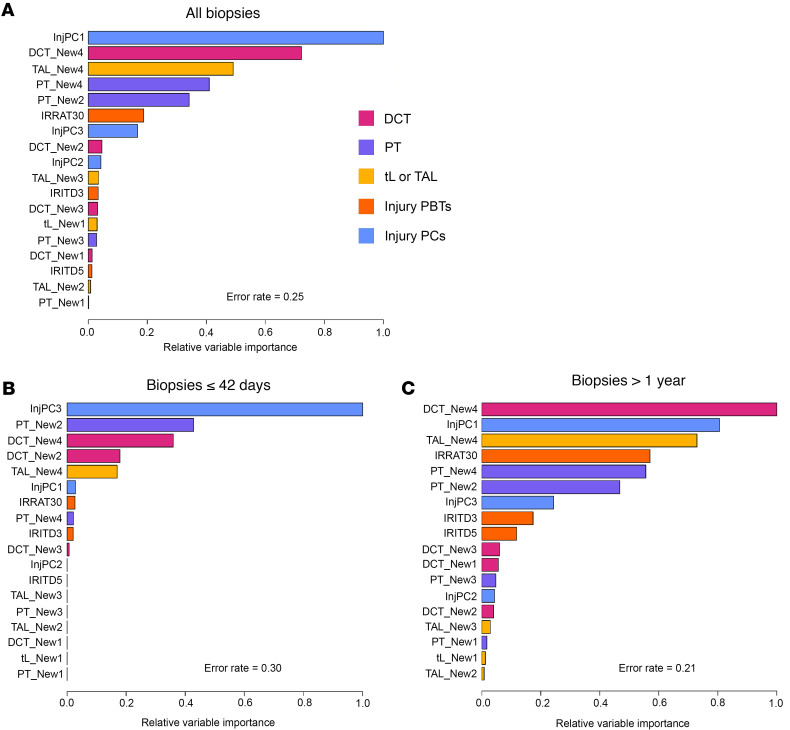
Relative variable importance plots for predicting 3-year survival after biopsy, using injury pathogenesis-based transcript set scores and injury principal component scores (using 1 randomly selected biopsy per transplant). (**A**) All 4,502 biopsies. (**B**) Biopsies >1 year. (**C**) Biopsies ≤42 days. Error rates are 1.0 minus the C statistic (which is the survival analysis equivalent of the AUC).

We used random survival forests to examine the relative importance of the 12 new injury gene sets for predicting 3-year death-censored survival in kidney transplants (1,292 of the 4,502 kidney biopsies with available follow-up data; 1 random biopsy per transplant) (Figure 8). The forests included kidney transplant–derived injury measurements (injury PC scores and gene sets). In all biopsies, the best predictors of 3-year survival were injury PC1 and the New4 scores representing EMT (Figure 8A). Injury PC3 also affected survival. In kidneys having early biopsies, PC3 was the dominant predictor, but the new injury transcript sets also had some impact (Figure 8B). Findings in late biopsies (>1 year posttransplant, Figure 8C) were similar to findings in all biopsies.

### Correlations of selected individual new injury genes in the kidney transplant injury model.

We examined the correlations of a sample of 13 new injury genes selected from the native kidney AKI studies in the kidney transplant model (Supplemental Table 10). These genes were selected because they were mentioned in the original paper (16). The genes annotated in the New4 gene sets such as IFITM3, VCAM1, HIF1A, and MET had strong associations with injury PC1, but many also correlated with injury PC3, e.g., MET, SPP1, and MYO5B. Genes associated with New1 states, such as ALDOB, LRP2, and NQO1, had negative correlations with injury PC1 but positive correlations with PC3. For NQO1, LRP2, and MYO5B, the strongest positive correlations were with PC3.

## Discussion

This study characterized molecular injury in a large cohort of kidney transplant biopsies using injury scores previously defined in transplant studies and explored how gene sets defined in single failed repair cells in native kidneys correlated with the features of kidney transplants. In the transplant biopsies, molecular injury did not separate into discrete groups such as AKI and CKD, instead forming continuous gradients in 3 dimensions: PC1, PC2, and PC3. PC1 correlated with many AKI and CKD features and related inflammation; PC2 defined variance related to time; PC3 defined injury-induced epithelial remodeling and anticorrelated with inflammation. This transplant injury model allowed us to study how the failed repair states previously described in native kidneys with AKI mapped in a large cohort of transplants. Expression of the new failed repair gene sets in transplants was consistent with their description as biological processes in native kidneys and correlated strongly with the injury PC scores in the transplant biopsies. In particular, the New4 gene sets related to EMT were as strongly related to injury PC1, outcomes, dysfunction, and even donor age as any gene sets and classifiers previously derived in transplants. This illustrates how discoveries in single-nucleus transcriptome data that are necessarily limited to small numbers of samples can be integrated into a bulk transcriptomics model derived from large numbers of biopsies from a relevant population, combining the power of these 2 high-dimensionality approaches.

As summarized in Supplemental Table 13, the major finding in this study is that there are 2 major independent processes in the kidney response to injury: PC1, the typical response to recent injury, and PC3, which involves epithelial remodeling. PC1 combines AKI-induced and failed repair transcripts, macrophage infiltration, and mitosis and had an increasing effect on the risk of failure with time. Positive PC3 anticorrelates with inflammation and mitosis but is also correlated with expression of failed repair gene sets. PC1 and PC3 are independently associated with lower eGFR and older donor age. The independence of these elements is clear in the diversity they reveal within AKI and CKD: AKI2 had more positive PC1 than AKI1, but AKI1 had more positive PC3; CKDAKI had more positive PC1 and PC3 than mild CKD, but mild CKD had more immunoglobulin transcripts.

The striking association of the new gene sets representing failed repair with the injury dimensions in transplants, and the previous finding that differences between individuals in the native kidney studies were driven by the abundance of cells in the injury states (14), argues that failed repair epithelial cells accumulate in kidney transplants, at least those undergoing biopsies. (Admittedly, this must ultimately be shown in single-nucleus transcriptome studies in transplants.) The New4 gene sets representing failed repair and EMT correlated strongly with injury PC1 but also moderately with injury PC3. Each set tended to behave like others in its new designation with no allegiance to the nephron segment of origin. The strength of the correlations of the New4-EMT gene sets with injury PC1, AKI2, CKDAKI, low eGFR, progression to failure, and donor age support the conclusion that failed repair and EMT are strongly associated with adverse transplant outcomes. However, New1 gene sets were protective, correlating with lower risk and higher eGFR, with minimal correlations with donor age.

The correlation of PC3 with CKDAKI, donor age, dysfunction, outcomes, and expression of all New4 gene sets suggests that PC3 (conditioned in part by donor age) is an element independent of injury PC1 that correlates with the added risk of graft loss at all times posttransplant (14, 16) but may be a response to failed repair that attempts to restore nephron integrity when missing cells cannot be replaced. PC3 correlates with injury-induced increased expression of genes involved in epithelial development and remodeling, independent of PC1 mathematically (by definition in PCA) and presumably biologically. PC3 is the dominant influence on risk in biopsies in the first weeks posttransplant when the relative impact of PC1 is minimal. In this formulation, optimal recovery from injury requires effective repair; aging increases failed repair and requires more PC3. The correlations of the new gene sets in transplants with PC3 (epithelial remodeling), donor age, dysfunction, and risk of failure argue that these associations should be explored in native kidneys.

These findings suggest a theoretical model of response to injury that may help design future experimental studies. Injury evokes a repair response that requires mitosis, evokes inflammation, and persists in a variety of failed repair cells (PC1) but also evokes epithelial remodeling without mitosis, particularly when repair is limited. Incomplete repair over time creates nephron loss and fibrosis (mild CKD). However, the persistence of failed repair with EMT-related changes and inflammation (perhaps triggered by secretory failed repair cells) leads to CKDAKI, with increased PC3/epithelial remodeling. The adverse associations of failed repair are underscored by the high correlation of New4 gene sets with PC1, CKDAKI, dysfunction, risk of failure, and donor age.

A key issue that cannot be resolved in biopsy-based cross-sectional studies is how the early responses to injuries at the time of transplantation relate to the changes observed in late biopsies years or decades later in the population. It is neither ethical nor practical to perform protocol biopsies in a decades-long time series: biopsies are potentially complicated interventions, and our study protocol specified standard-of-care biopsies. It seems likely the AKI transcripts and new gene sets are expressed in many injured cells, with peak expression occurring 3–5 days posttransplant in our mouse kidney transplant isograft model and in other studies (13), followed by declining expression as much of the injury is successfully repaired (3) but then increasing in the late biopsy population. The time courses of the expression of the New4-EMT transcripts sets is similar to that of the AKI-induced transcripts identified in transplants, and their expression in late biopsies probably reflects the accumulation of failed repair cells, but whether the rise with time is a late consequence of early injury or an indication of new injury cells is not known (13, 14, 16). However, the increasing association of PC1 with risk of failure, even in biopsies with no rejection, makes these questions very relevant.

The limitations of this study include its cross-sectional design in the population of biopsies for clinical indications per standard of care and its use of microarrays rather than RNA sequencing. Every biopsy from a consented patient was included, but the best kidney transplants never get biopsied, limiting generalization from biopsy-based observations to the unbiopsied population. We note that other donor or recipient features may also contribute to graft function and failure. We cannot fully exclude all effects of rejection even in biopsies called no rejection in these analyses because rejection can operate at subthreshold levels,and could have been operating at earlier times to induce the injury observed in the present biopsies (23, 25, 26). Finally, while the new gene sets were annotated in single failed repair epithelial cells, we acknowledge that we do not formally know whether the expression of the new gene sets in the bulk transcriptomics model reflects the accumulation of single failed repair epithelial cells and whether other cells such as endothelial or matrix cells express these genes.

We acknowledge that RNA sequencing is the ideal platform for discovery, but we selected microarrays because our goal is to apply the MMDx system in diagnostic service laboratories that are clones of the discovery laboratory, using the algorithms defined in the discovery laboratory. We found that microarrays are ideal for this purpose. Their high standardization, manufacturing quality, and mature analysis strategies are advantageous when performing genome-wide molecular analysis in multiple sites, and the strong correlation of gene sets derived in single-nucleus RNA sequencing with the features of the bulk transcriptomic transplant model is reassuring. The microarray measurements in a remote service laboratory, such as Kashi Clinical Laboratories (see Figure 1), are virtually identical to those in the discovery laboratory, which is essential for the application of the many machine learning algorithms developed in the Edmonton discovery laboratory.

Despite their associations, we are reluctant to call PC1/failed repair or PC3 maladaptive until we can abrogate these elements and determine the consequences. The response to wounding is an ancient and ultimately beneficial response of all multicellular organisms, even if blunted by the aging process, but it still may be better than failure to respond. The real maladaptive elements are the agents causing injury, which we must strive to eliminate, even as we seek opportunities to mitigate their consequences. Whether failed repair cells are innocent bystanders or injurious will have to be shown by interventions that manipulate the cells and change outcomes. Failed repair cells may reflect the net failure of regeneration, with loss of nephron reserve and capacity for peak function, exposing the remaining cells to the possibility of exhaustion. However, some failed repair cells may actively interfere with nephron function and evoke more injury, and trigger EMT, further increasing the number of failed repair cells and ultimately driving nephron loss. As we noted above, the inflammation in CKDAKI may reflect the secretory properties of failed repair cells, analogous to the impact of senescent cells (16). The possibility that failed repair cells are actively harmful like senescent cells opens the possibility of senolysis-like treatments to mitigate the adverse impact of failed repair cells (27). Focusing on individual molecules mapped in the present study could also be useful. For example, chemokine CXCL6 is not induced by AKI but is strongly associated with CKD in transplants (24) and with EMT generally (28) and is of interest because it has not been a feature of failed repair epithelial cells. Thus, these studies of injury in transplants and in native kidneys open many new opportunities for experimental exploration.

## Methods

### Sex as a biological variable.

This study includes both male and female kidney transplant recipients and donors and did not exclude any biopsies based on recipient or donor sex to represent the true transplant population as closely as possible. Similar to the overall registered kidney transplant population (29), we found an overrepresentation of male recipients (62% male recipients). Sex was not considered as a biological variable for these analyses, as we focused primarily on population-wide analyses.

### Patient population and data collection.

The patient and biopsy population were similar to previous studies (7, 23). We used all valid/available research biopsies and accompanying phenotype data collected as part of the MMDx-Kidney studies (INTERCOMEX NCT01299168, and Trifecta-Kidney NCT04239703) (22, 23, 30–36). Data transmitted from the local centers for analysis at the ATAGC were sent via electronic or printed forms and stored in a REDCap database. Recruitment for this study began in September 2008 and was extended to May 2022, mean follow-up time was 858 days (range 1–10.5 years), and data collection was completed on September 15, 2024.

The 5,086 biopsies were assessed by microarrays (23) and had complete gene expression data (49,495 probe set values representing 19,462 genes). MMDx diagnoses sign-outs were available for most of the data set. For the present injury analysis, we included only biopsies with more than 10% cortex (37) (resulting in the final population of 4,502 biopsies), but including all biopsies created similar models. Quantitative variables in these analyses included injury archetype scores (2); PBT scores, which include the new injury gene sets (14) (Supplemental Table 1); classifier scores (Supplemental Table 2); and principal component scores (Figure 2). Quantitative variables were used as continuous values except where noted. Archetype groups were assigned based on the highest archetype score for that biopsy, as previously described (2, 22), to obtain meaningful groupings based on molecular data.

### Biopsy processing.

As previously described (12), a portion of 1 core of each biopsy (mean length 3 mm) (33) was immediately stabilized in RNA*later* (Thermo Fisher Scientific) and shipped to the ATAGC (http://atagc.med.ualberta.ca) or Kashi Clinical Laboratories at ambient temperature for RNA extraction and processing as previously described (33). Gene expression was measured using GeneChip PrimeView 219 Human Gene Expression Arrays (Applied Biosystems). All molecular diagnoses were made without knowledge of the biopsy’s corresponding histology, clinical data, or human leukocyte antibody status. MMDx reports were sent to the participating centers, usually within 2 working days of receiving the biopsy.

### Transcript sets.

PBTs are described in Supplemental Table 1 and the ATAGC home page (https://www.ualberta.ca/medicine/institutes-centres-groups/atagc/research/gene-lists.html). The new transcript sets (14) are detailed in Supplemental Table 1. PBT scores are calculated as the mean fold-change in expression across all probe sets in the PBT versus the mean expression of the same probe sets in a control group (4 nephrectomy samples).

### Updating the published injury classifiers.

The probabilities of histologic atrophy and fibrosis, low GFR, and proteinuria were updated in the new, larger *N* = 4,502 data set using the same methodology as previously published (22, 32). We rederived these 4 classifiers (Supplemental Table 2) using the ClinicalTrials.gov biopsies that had the appropriate phenotype recorded: (eGFR ≤ 30 cc/min/M^2^; lowGFR_Prob_), proteinuria positivity (Prot_Prob_), fibrosis ci lesion scores (ci>1_Prob_), and atrophy ct lesion scores > 1 (ct>1_Prob_). For classifier development, PCA, AA, and UMAP, we excluded biopsies with estimated cortex content less than 10% (37), since the reliability of some injury-related molecular readings is slightly more variable in samples that are predominantly medulla (37). The full classifier process is outlined in Figure 1B. AUC for the binary classes used for training classifiers (Supplemental Table 2) ranges from 0.68 (proteinuria) to 0.86 (GFR). The low AUCs for the proteinuria classifier (0.68) probably reflect the different standard-of-care methods and definitions in the centers. Note that the purpose of these classifiers trained on the conventional and histologic labels is not to predict the class used for training — this is already known — but to find the molecular states associated with these labels, taking advantage of the large *N* of samples and the high information content of the microarray chip. For example, the Prot_Prob_ classifier assigns the probability that the biopsy has molecular changes that correlate with proteinuria, whether they have been designated as positive for proteinuria or not.

### Rejection classifiers.

The rejection-based PCA/AA and the 7 classifiers used to build them in the 5,086 set have been published previously (23). Figure 1B uses these rejection scores, only showing the distribution in the 4,502 biopsies with an estimated %cortex ≥ 10% used in this study.

### Injury AA, PCA, and UMAP.

Six previously defined injury-related PBT scores (Supplemental Table 1) and 4 newly updated injury classifier scores (Supplemental Table 2) were used as inputs for both the injury PCA and the injury AA. Each biopsy was assigned 5 archetype scores reflecting its proximity to each archetype center and assigned to 1 AA group based on its highest archetype score. UMAP (38) was used as an alternative to PCA for dimensionality reduction and visualization.

### Survival analysis.

Survival was defined as death-censored graft loss by 3 years after biopsy, using time of biopsy as time 0, and selecting 1 random biopsy per transplant for analysis from the samples that had follow-up data (*N* = 1,292 biopsies, with 210 failures by 3 years postbiopsy). Random survival forests used the randomForestSRC (39) package with default parameter settings other than importance=“permute”, ntree=5000, nsplit=1, and na.action=“na.impute.” Error rates were defined as 1.0 – the C statistic, as measured in the out-of-bag samples in the forests. Relative variable importance was determined using rfsrc’s permutation method. Kaplan-Meier plots were generated using the survival (40) and survminer (41) packages. The hazard of graft loss was estimated from a Cox proportional hazards model with interaction terms between time posttransplant and the injury PCs using the coxph function from the survival (40) package.

### Cell panel.

The tissue and cell basis of expression of each gene was interpreted using microarray results of a previously studied cell panel (44), as well as literature and the Human Protein Atlas (45).

### Statistics.

All analyses were performed with the R computing language (42) version 4.3.2. Restricted cubic splines with 3 knots were generated using the rms package (43). *P* < 0.05 was considered statistically significant.

### Study approval.

Histologic scores, diagnoses, clinical data, and DSA status were assigned by the local center’s standard of care following Banff guidelines. Biopsy collection was approved by IRBs at each center (locations given in the list of author institutions for this manuscript) and approved by the central IRB (Research Ethics Office) at the University of Alberta, Edmonton, Alberta, Canada (Pro00022226). Written informed consent was obtained from all patients before enrollment as approved by the local center’s IRB.

### Data availability.

CEL files are available on the NCBI’s Gene Expression Omnibus (accession number GSE275126). The Supporting Data Values file is available in the supplement.

## Author contributions

PFH was the principal investigator, edited and reviewed the manuscript, and was responsible for data interpretation and study design. JC was responsible for data analysis and reviewing the manuscript. M Mackova was responsible for biopsy processing and manuscript reviewing. KSMT was responsible for editing and reviewing the manuscript. PTG was responsible for data interpretation and editing and reviewing the manuscript. CH was responsible for data interpretation and reviewing the manuscript. GB, JB, KB, GE, FE, GG, M Myślak, OV, EA, TA, SA, MA, RB, MB, CDB, DB, AC, KC, MC, DDF, DDM, ADS, AD, LD, MD, RF, IF, JF, John Gill, Jagbir Gill, M Glyda, SG, M Gryczman, P Hruba, P Hughes, AJ, ZJ, LK, M Kamel, SK, NK, JK, JL, RBM, AM, JM, M Miglinas, TM, SN, BN, AP, APP, MP, GP, EP, SRB, TS, SS, MLNS, JS, KSJ, ZVH, KV, MRW, AW, ZW, HY, and ZZ were responsible for biopsy sample collection and manuscript reviewing.

## Supplementary Material

Supplemental data

ICMJE disclosure forms

Supporting data values

## Figures and Tables

**Table 8:**
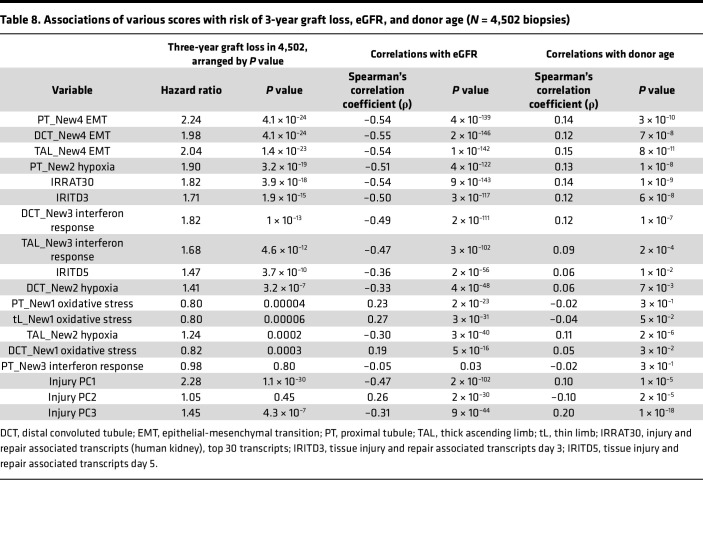
Associations of various scores with risk of 3-year graft loss, eGFR, and donor age (*N* = 4,502 biopsies)

**Table 7:**
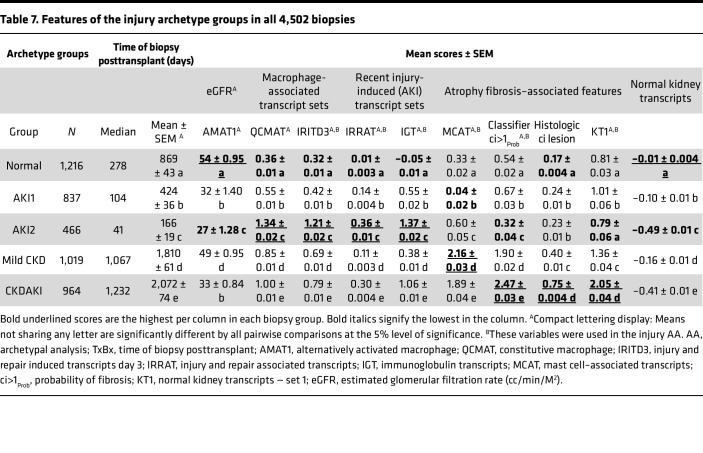
Features of the injury archetype groups in all 4,502 biopsies

**Table 6:**
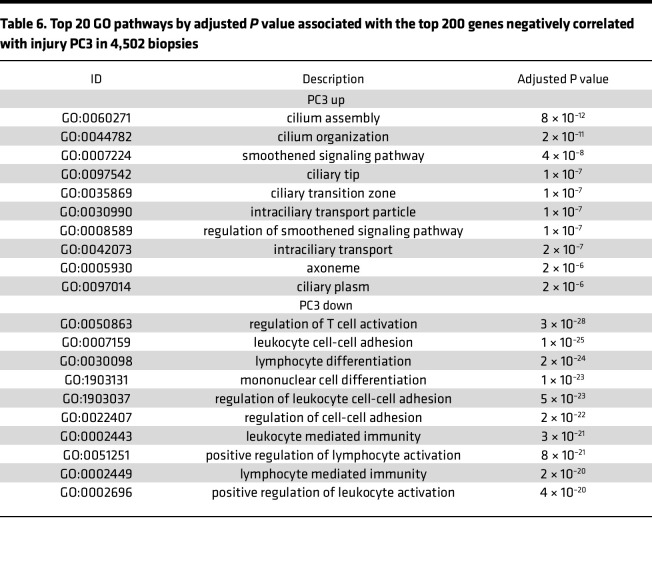
Top 20 GO pathways by adjusted *P* value associated with the top 200 genes negatively correlated with injury PC3 in 4,502 biopsies

**Table 5:**
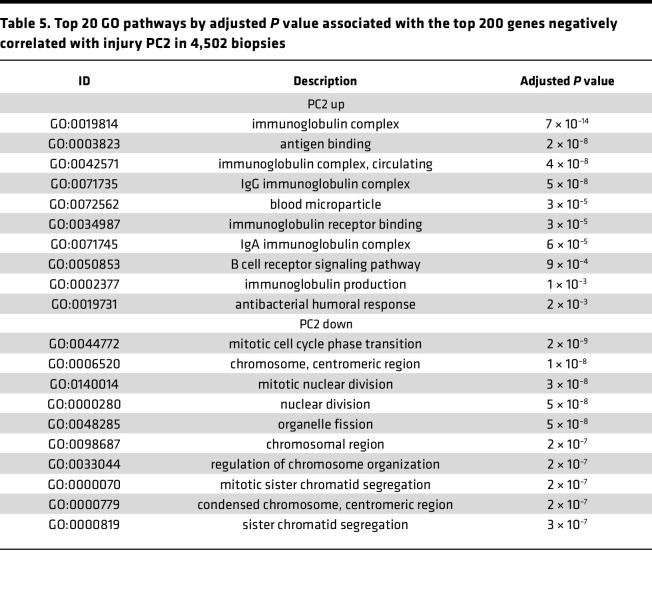
Top 20 GO pathways by adjusted *P* value associated with the top 200 genes negatively correlated with injury PC2 in 4,502 biopsies

**Table 4:**
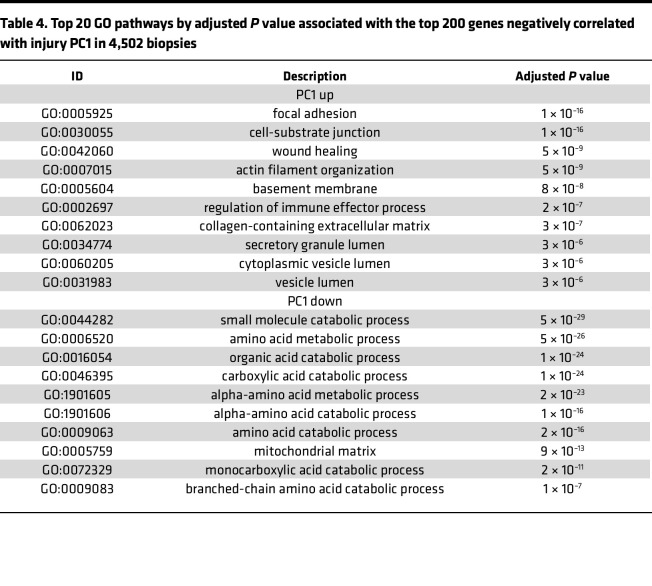
Top 20 GO pathways by adjusted *P* value associated with the top 200 genes negatively correlated with injury PC1 in 4,502 biopsies

**Table 3:**
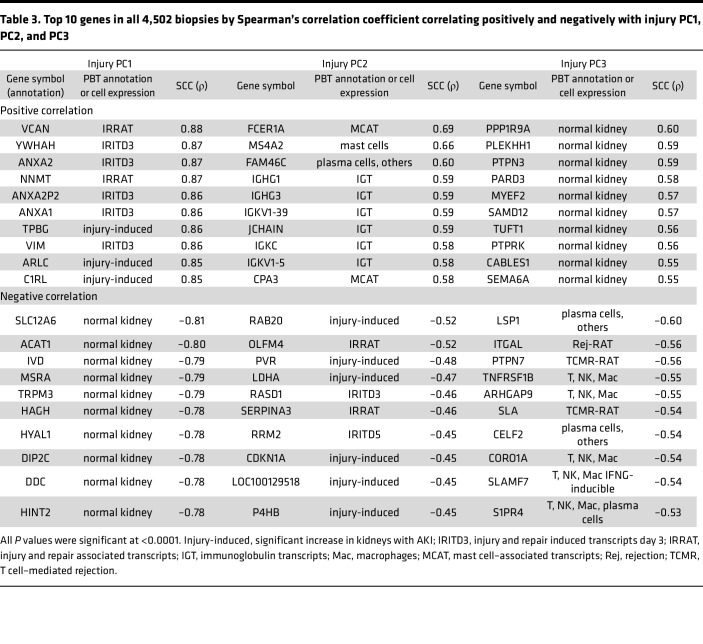
Top 10 genes in all 4,502 biopsies by Spearman’s correlation coefficient correlating positively and negatively with injury PC1, PC2, and PC3

**Table 1:**
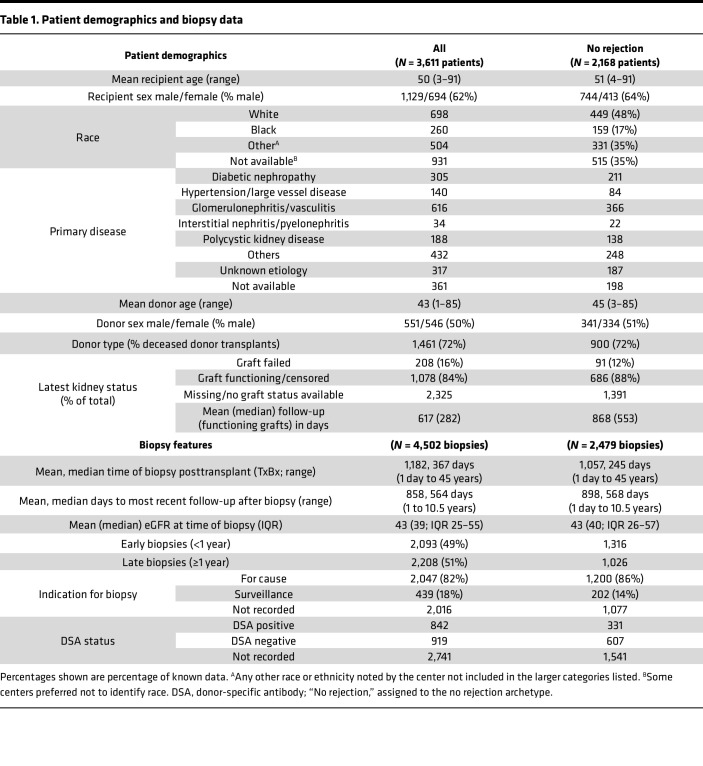
Patient demographics and biopsy data

**Table 2:**
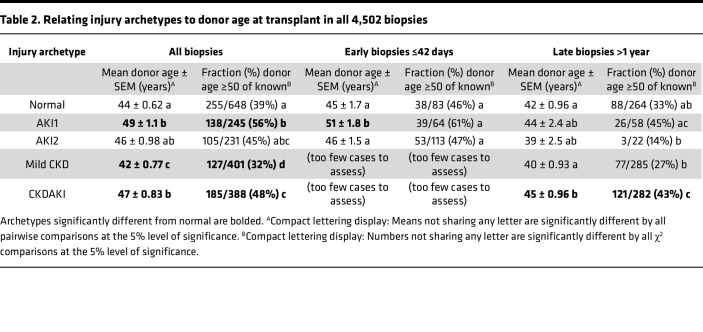
Relating injury archetypes to donor age at transplant in all 4,502 biopsies

## Version 1 04/15/2025

In-Press Preview

## Version 2 05/22/2025

Electronic publication
