# Supplementary material for: A cross-sectional study of the role of epithelial cell injury in kidney transplant outcomes
Source: JCI Insight. 2025 Apr 15;10(10):e188658. doi: 10.1172/jci.insight.188658 (PMC12128995; doi:10.1172/jci.insight.188658)
Supplement: Supplemental data [file jciinsight-10-188658-s101.pdf]

A cross-sectional study of the role of epithelial cell injury in kidney transplant outcomes

Philip F Halloran[1, 2,*], Jessica Chang[1], Martina Mackova[1], Katelynn S. Madill-Thomsen[1], Enver Akalin[3], Tarek Alhamad[4], Sanjiv Anand[5], Miha Arnol[6], Rajendra Baliga[7], Mirosław Banasik[8], Christopher D. Blosser[9], Georg Böhmig[10], Daniel Brennan[11], Jonathan Bromberg[12], Klemens Budde[13], Andrzej Chamienia[14], Kevin Chow[15], Michał Ciszek[16], Declan de Freitas[17], Dominika Dęborska-Materkowska[18], Alicja Debska-Ślizień[14], Arjang Djamali[18], Leszek Domański[19], Magdalena Durlik[16], Gunilla Einecke[20], Farsad Eskandary[10], Richard Fatica[21], Iman Francis[22], Justyna Fryc[23], John Gill[24], Jagbir Gill[24], Maciej Glyda[25], Sita Gourishankar[2], Marta Gryczman[19], Gaurav Gupta[26], Petra Hruba[27], Peter Hughes[15], Arskarapurk Jittirat[28], Zeljka Jurekovic[29], Layla Kamal[26], Mahmoud Kamel[7], Sam Kant[11], Nika Kojc[30], Joanna Konopa[14], James Lan[24], Roslyn B. Mannon[31], Arthur Matas[32], Joanna Mazurkiewicz[19], Marius Miglinas[33], Thomas Mueller[34], Marek Myślak[19], Seth Narins[35], Beata Naumnik[23], Anita Patel[22], Agnieszka Perkowska-Ptasińska[16], Michael Picton[17], Grzegorz Piecha[36], Emilio Poggio[21], Silvie Rajnochová Bloudíčkova[27], Thomas Schachtner[34], Soroush Shojai[2], Majid L.N. Sikosana[2], Janka Slatinská[27], Katarzyna Smykal-Jankowiak[25], Ashish Solanki[11], Željka Veceric Haler[30], Ondrej Viklicky[27], Ksenija Vucur[29], Matthew R. Weir[12], Andrzej Wiecek[36], Zbigniew Włodarczyk[37], Harold Yang[35], Ziad Zaky[21], Patrick T. Gauthier[1,†], and Christian Hinze[38,†]

[1]Alberta Transplant Applied Genomics Centre; [2]University of Alberta, Edmonton, AB, Canada; [3]Montefiore Medical Center, Bronx, NY, USA; [4]Washington University at St. Louis, St. Louis, MO, USA; [5]Intermountain Transplant Services, Murray, UT, USA; [6]University of Ljubljana, Ljubljana, Slovenia; [7]Tampa General Hospital, Tampa, FL, USA; [8]Medical University of Wrocław, Wrocław. Poland; [9]University of Washington, Seattle, WA, USA; [10]Medical University of Vienna, Vienna, Austria; [11]Johns Hopkins University School of Medicine, Baltimore, MD, USA; [12]University of Maryland, Baltimore, MD, USA; [13]Charite-Medical University of Berlin, Berlin, Germany; [14]Medical University of Gdańsk, Gdańsk, Poland; [15]The Royal Melbourne Hospital, Parkville, Australia; [16]Warsaw Medical University, Warsaw, Poland; [17]Manchester Royal Infirmary, Manchester, UK; [18]University of Wisconsin, Madison, WI, USA; [19]Pomeranian Medical University, Szczecin, Poland; [20]Medical University of Hannover, Hannover, Germany; [21]Cleveland Clinic Foundation, Cleveland, OH, USA; [22]Henry Ford Transplant Institute, Detroit, MI, USA; [23]Medical University in Bialystok, Białystok, Poland; [24]St. Paul's Hospital, Vancouver, BC, Canada; [25]Wojewodzki Hospital, Poznan, Poland; [26]Virginia Commonwealth University, Richmond, VA, USA; [27]Institute for Experimental and Clinical Medicine, Prague, Czech Republic; [28]University Hospital Cleveland Medical Center, Cleveland, OH, USA; [29]University Hospital Merkur, Zagreb, Croatia; [30]University of Ljubljana, Ljubljana, Slovenia; [31]University of Alabama at Birmingham, Birmingham, AL, USA; [32]University on Minnesota, Minneapolis, MN, USA; [33]Vilnius University Hospital Santaros Klinikos, Vilnius, Lithuania; [34]University Hospital Zurich, Zurich, Switzerland; [35]PinnacleHealth Transplant Associates, Harrisburg, PA, USA; [36]Silesian Medical University, Katowice, Poland; [337]University Hospital no. 1, Bydgoszcz, Poland; [38]Hannover Medical School, Hannover, Germany;

INTERCOMEX Study: ClinicalTrials.gov #NCT01299168; **Trifecta-Kidney Study**: ClinicalTrials.gov NCT01299168

*Corresponding author

†Co-senior authors

Supplemental Table 1. Description of pathogenesis-based transcript sets (PBTs)[A]

Category		Abbreviation	Description
Injury	Recent injury-related	IRITD3[B]	Injury-repair induced, day 3 (IRITD3) (1)
		IRITD5[B]	Injury-repair induced, day 5 (IRITD3) (1)
		IRRAT[B]	Injury-repair associated (IRRAT) (2)
	Late injury (atrophy-fibrosis)-related	IGT[B]	Immunoglobulin transcripts (IGT) (3)
	Normal kidney transcripts	KT1	Normal kidney transcripts-set 1 (4)
		KT2	Normal kidney transcripts-set 2 (4)
	Macrophage-related	AMAT1	Alternatively activated macrophage (AMAT1) (5)
		QCMAT	Constitutive macrophage (QCMAT) (5)
	Damage-associated	DAMPs[B]	Damage associated molecular patterns (6)
	Inflammation	MCAT[B]	Mast cell transcripts (7)
Derived in human kidneys with AKI	“New” injury gene sets	PT_New1	Proximal tubule oxidative stress (8, 9)
		tL_New1	Thin limb oxidative stress (8, 9)
		DCT_New1	Distal convoluted tubule oxidative stress (8, 9)
		PT_New2	Proximal tubule hypoxia (8, 9)
		TAL_New2	Thick ascending limb hypoxia (8, 9)
		DCT_New2	Distal convoluted tubule hypoxia (8, 9)
		PT_New3	Proximal tubule interferon response (8, 9)
		TAL_New3	Thick ascending limb interferon response (8, 9)
		DCT_New3	Distal convoluted tubule interferon response (8, 9)
		PT_New4 EMT	Proximal tubule epithelial mesenchymal transition (8, 9)
		TAL_New4 EMT	Thick ascending limb epithelial mesenchymal transition (8, 9)
		DCT_New4 EMT	Distal convoluted tubule epithelial mesenchymal transition (8, 9)

[A] https://www.ualberta.ca/medicine/institutes-centres-groups/atagc/research/gene-lists

[B] PBTs used in injury archetypal analysis

References:

1. Famulski KS, Broderick G, Einecke G, Hay K, Cruz J, Sis B et al. Transcriptome analysis reveals heterogeneity in the injury response of kidney transplants. Am J Transplant 2007;7(11):2483-2495.
2. Famulski KS, de Freitas DG, Kreepala C, Chang J, Sellares J, Sis B et al. Molecular phenotypes of acute kidney injury in kidney transplants. J Am Soc Nephrol 2012;23(5):948-958.
3. Einecke G, Reeve J, Mengel M, Sis B, Bunnag S, Mueller TF et al. Expression of B cell and immunoglobulin transcripts is a feature of inflammation in late allografts. Am J Transplant 2008;8(7):1434-1443.
4. Einecke G, Broderick G, Sis B, Halloran PF. Early loss of renal transcripts in kidney allografts: relationship to the development of histologic lesions and alloimmune effector mechanisms. Am J Transplant. 2007;7(5):1121-30.
5. Famulski KS, Einecke G, Sis B, Mengel M, Hidalgo LG, Kaplan B et al. Defining the canonical form of T-cell-mediated rejection in human kidney transplants. Am J Transplant 2010;10(4):810-820.
6. Land WG, Agostinis P, Gasser S, Garg AD, Linkermann A: Transplantation and Damage-Associated Molecular Patterns (DAMPs). Am J Transplant 2016;16:3338-61.
7. Mengel M, Reeve J, Bunnag S, Einecke G, Sis B, Mueller T, et al. Molecular correlates of scarring in kidney transplants: the emergence of mast cell transcripts. Am J Transplant. 2009;9(1):169-78.
8. Hinze C, Kocks C, Leiz J, Karaiskos N, Boltengagen A, Cao S, et al. Single-cell transcriptomics reveals common epithelial response patterns in human acute kidney injury. Genome Med. 2022;14(1):103.
9. Hinze C, Lovric S, Halloran PF, Barasch J, and Schmidt-Ott KM. Epithelial cell states associated with kidney and allograft injury. Nat Rev Nephrol. 2024;20(7):447-59.

Supplemental Table 2. Classifiers used and respective AUCs in all 4502 biopsies (with known phenotyping)

Classifier	AUC	Positive (bad) class	Negative class	Not recorded[A]
$ci>1_{Prob}$	0.81	675	1253	1197
$ct>1_{Prob}$	0.83	564	1364	1197
$lowGFR_{Prob}$	0.87	629	1225	1271
$Prot_{Prob}$	0.68	1074	763	1288

[A] Out of 3125 with cortex>10% and with known scores
Abbreviations: $ci>1_{Prob}$ - probability of histologic fibrosis; $ct>1_{Prob}$ - probability of histologic atrophy; $lowGFR_{Prob}$ – probability of low GFR≤30cc/min/M2; $Prot_{Prob}$ - probability of proteinuria; AUC, area-under-the-curve

Supplemental Table 3. Spearman correlations of PC scores with donor age in 1913 biopsies (with donor age recorded)[A]

PC score	Spearman correlation coefficient (ρ)	*P* value
**Injury PC1**	0.10	$1x10^{-5}$
**Injury PC2**	-0.10	$2x10^{-5}$
**Injury PC3**	0.20	$<2x10^{-16}$

[A] from 4502 biopsies with cortex>10% and recorded donor age
Abbreviations: PC, principal component; PC1/2/3 – principal components 1/2/3

Supplemental Table 4. Top 10 genes in 2479 No Rejection biopsies by Spearman correlation coefficient correlating positively and negatively with injury PC1, PC2, and PC3[A]

Injury PC1			Injury PC2			Injury PC3		
**Gene symbol**	**PBT annotation or cell expression**	**SCC (ρ)**	**Gene symbol**	**PBT annotation or cell expression**	**SCC (ρ)**	**Gene symbol**	**PBT annotation or cell expression**	**SCC (ρ)**
Positive correlation								
VIM	injury-induced	*0.88*	FCER1A	MCAT	*0.67*	PLEKHH1	normal kidney	*0.61*
VCAN	IRRAT	*0.87*	MS4A2	MCAT	*0.67*	KLF8	normal kidney	*0.58*
ANXA1	IRITD3	*0.87*	IGKC	IGT	*0.65*	PARD3	normal kidney	*0.58*
ANXA2	IRITD3	*0.87*	JCHAIN	IGT	*0.65*	PTPRK	normal kidney	*0.57*
TPBG	injury-induced	*0.87*	IGHG1	IGT	*0.64*	ZDHHC23	normal kidney	*0.57*
NNMT	IRRAT	*0.87*	IGHG3	IGT	*0.64*	USP43	normal kidney	*0.57*
ANXA2P2	IRITD3	*0.87*	IGKV1-5	IGT	*0.64*	NBEA	normal kidney	*0.56*
YWHAH	IRITD3	*0.86*	IGKV1-39	IGT	*0.64*	SAMD12	normal kidney	*0.56*
MMP7	IRITD5	*0.85*	IGKV1-27	IGT	*0.63*	TUFT1	normal kidney	*0.56*
TIMP1	injury-induced	*0.85*	IGKC	IGT	*0.63*	SEMA6A	normal kidney	*0.56*
Negative correlation								
SLC12A6	normal kidney	*-0.79*	OLFM4	IRRAT	*-0.56*	LSP1	Plasma cells, others	*-0.56*
TRPM3	normal kidney	*-0.77*	RAB20	injury-induced	*-0.54*	PTPN7	TCMR-RAT	*-0.52*
IVD	normal kidney	*-0.76*	SPP1	injury-induced	*-0.52*	RNF166	T, NK, Macrophage	*-0.51*
DIP2C	normal kidney	*-0.76*	SERPINA3	injury-induced	*-0.52*	ITGAL	Rejection-RAT	*-0.51*
MSRA	normal kidney	*-0.76*	RASD1	IRITD3	*-0.50*	IKZF1	T, NK, Macrophage	*-0.50*
AGMAT	normal kidney	*-0.75*	PVR	injury-induced	*-0.50*	CD7	T, NK	*-0.50*
HINT2	normal kidney	*-0.75*	ADAMTS1	IRRAT	*-0.49*	PRF1	T,NK,QCAT	*-0.50*
TMIGD1	normal kidney	*-0.75*	C2CD4A	injury-induced	*-0.49*	TNFRSF1B	T, NK, Macrophage	*-0.49*
DIO1	normal kidney	*-0.75*	RRM2	IRITD5	*-0.48*	ARHGAP4	T, NK, Macrophage	*-0.49*
TMIGD1	normal kidney	*-0.75*	P4HB	injury-induced	*-0.47*	ZAP70	T, NK	*-0.49*

[A] All *P* values were significant at <0.0001; injury-induced indicates significantly increased in kidneys with clinically-defined AKI.

Abbreviations: PC, principal component; PBT, pathogenesis-based transcript set; SCC, Spearman correlation coefficient, IRRAT, injury/repair associated transcripts (human kidney); IRITD3, tissue injury and repair associated transcripts; IRITD5, tissue injury and repair associated transcripts; MCAT, Mast cell-associated transcripts; IGT, immunoglobulin transcripts; TCMR-RAT, TCMR-associated RATs, NK, natural killer; T, T cells; Rejection-RAT, Rejection-associated RATs

Supplemental Table 5. Top 20 GO pathways by adjusted P value associated with the top 200 genes negatively correlated with Injury PC1, PC2 and PC3 in 2479 No rejection biopsies

PC1 up			PC2 up			PC3 up		
**ID**	**Description**	**Adjusted *P* value**	**ID**	**Description**	**Adjusted *P* value**	**ID**	**Description**	**Adjusted *P* value**
GO:0002253	activation of immune response	6E-08	GO:0002377	immunoglobulin production	1E-06	GO:0044782	cilium organization	5E-05
GO:0034341	response to type II interferon	1E-07	GO:0050853	B cell receptor signaling pathway	3E-05	GO:0060271	cilium assembly	5E-05
GO:0006909	phagocytosis	2E-07	GO:0002440	production of molecular mediator of immune response	3E-05	GO:0007224	smoothened signaling pathway	6E-03
GO:0007015	actin filament organization	4E-07	GO:0050851	antigen receptor-mediated signaling pathway	1E-04	GO:0051960	regulation of nervous system development	1E-02
GO:0002697	regulation of immune effector process	9E-07	GO:0002757	immune response-activating signaling pathway	5E-04	GO:0001822	kidney development	1E-02
GO:0042060	wound healing	1E-06	GO:0002253	activation of immune response	7E-04	GO:0072001	renal system development	2E-02
GO:0019221	cytokine-mediated signaling pathway	2E-06	GO:0002764	immune response-regulating signaling pathway	8E-04	GO:0005912	adherens junction	3E-04
GO:0071346	cellular response to type II interferon	7E-06	GO:0002443	leukocyte mediated immunity	8E-04	GO:0005930	axoneme	1E-03
GO:0071222	cellular response to lipopolysaccharide	2E-05	GO:0002460	adaptive immune response based on somatic recombination of immune receptors built from immunoglobulin superfamily domains	9E-04	GO:0097014	ciliary plasm	1E-03
GO:0002443	leukocyte mediated immunity	3E-05	GO:0001909	leukocyte mediated cytotoxicity	9E-04	GO:0005881	cytoplasmic microtubule	3E-03
**PC1 down**			**PC2 down**			**PC3 down**		
**ID**	**Description**	**Adjusted *P* value**	**ID**	**Description**	**Adjusted *P* value**	**ID**	**Description**	**Adjusted *P* value**
GO:0044282	small molecule catabolic process	3E-25	GO:0044772	mitotic cell cycle phase transition	9E-05	GO:1903131	mononuclear cell differentiation	8E-07
GO:0006520	amino acid metabolic process	7E-24	GO:2001233	regulation of apoptotic signaling pathway	5E-03	GO:0030098	lymphocyte differentiation	2E-06
GO:0016054	organic acid catabolic process	3E-21	GO:0048144	fibroblast proliferation	5E-03	GO:0046631	alpha-beta T cell activation	2E-04
GO:0046395	carboxylic acid catabolic process	3E-21	GO:2001242	regulation of intrinsic apoptotic signaling pathway	5E-03	GO:0001909	leukocyte mediated cytotoxicity	9E-04
GO:1901605	alpha-amino acid metabolic process	4E-21	GO:0007093	mitotic cell cycle checkpoint signaling	5E-03	GO:0030217	T cell differentiation	1E-03
GO:1901606	alpha-amino acid catabolic process	7E-14	GO:0030198	extracellular matrix organization	5E-03	GO:0046632	alpha-beta T cell differentiation	1E-03
GO:0009063	amino acid catabolic process	7E-14	GO:0043062	extracellular structure organization	5E-03	GO:0043367	CD4-positive, alpha-beta T cell differentiation	1E-03
GO:0072329	monocarboxylic acid catabolic process	1E-11	GO:0045229	external encapsulating structure organization	5E-03	GO:0007159	leukocyte cell-cell adhesion	1E-03
GO:0006631	fatty acid metabolic process	7E-07	GO:0090399	replicative senescence	6E-03	GO:0002366	leukocyte activation involved in immune response	2E-03
GO:0009083	branched-chain amino acid catabolic process	5E-06	GO:0046718	viral entry into host cell	6E-03	GO:0002263	cell activation involved in immune response	3E-03

Supplemental Table 6. Top 10 genes correlating in 2479 No Rejection biopsies by Spearman correlation coefficient correlating positively and negatively with Injury archetype scores[A]

Normal			AKI1			AKI2			Mild CKD			CKDAKI		
**Gene symbol**	**PBT annotation or cell expression**	**SCC (ρ)**	**Gene symbol**	**PBT annotation or cell expression**	**SCC (ρ)**	**Gene symbol**	**PBT annotation or cell expression**	**SCC (ρ)**	**Gene symbol**	**PBT annotation or cell expression**	**SCC (ρ)**	**Gene symbol**	**PBT annotation or cell expression**	**SCC (ρ)**
Positive correlation														
TRPM3	normal kidney	*0.70*	ZDHHC23	normal kidney	*0.58*	SLC16A3	injury-induced	*0.57*	IGKV1-39	IGT	*0.63*	LAMC2	atrophy-fibrosis/AKI	*0.74*
HINT2	normal kidney	*0.70*	MTMR2	Injury-induced	*0.57*	CLIC1	IRITD3	*0.57*	JCHAIN	IGT	*0.63*	CXCL6	atrophy-fibrosis/AKI	*0.74*
AMT	normal kidney	*0.69*	SPTBN1	injury-induced	*0.55*	RAB13	Injury-induced	*0.56*	IGHG1	IGT	*0.63*	CXCL1	atrophy-fibrosis/AKI	*0.72*
CLCN5	normal kidney	*0.69*	BRE	normal kidney	*0.54*	SERPINA3	IRRAT	*0.56*	IGHG3	IGT	*0.63*	MMP7	IRITD5	*0.71*
RALYL	normal kidney	*0.68*	PARD3	normal kidney	*0.54*	SERPINH1	IRITD5	*0.56*	IGKV1-27	IGT	*0.63*	CCND2	injury-induced	*0.70*
GGT6	normal kidney	*0.68*	ACLY	Injury-induced	*0.52*	ADAMTS1	IRRAT	*0.56*	IGKV1-5	IGT	*0.62*	CPA3	MCAT	*0.69*
GLYCTK	normal kidney	*0.68*	OSBPL10	normal kidney	*0.52*	SOCS3	injury-induced	*0.55*	IGK	IGT	*0.62*	ANXA3	IRITD3	*0.69*
GLTPD2	normal kidney	*0.68*	BEND7	normal kidney	*0.51*	ARPC1B	IRITD3	*0.55*	IGKC	IGT	*0.62*	PROM1	atrophy-fibrosis/AKI	*0.69*
GGT6	normal kidney	*0.68*	MRRF	normal kidney	*0.51*	S100A9	QCMAT	*0.55*	IGKV3-11	IGT	*0.58*	ANXA1	IRITD3	*0.69*
DIP2C	normal kidney	*0.68*	KIF21A	normal kidney	*0.51*	SCARB2	injury-induced	*0.55*	IGLV1-41	IGT	*0.58*	TPSAB1	atrophy-fibrosis/AKI	*0.69*
Negative correlation														
TIMP1	Injury-induced	*-0.81*	IGLL3P	IGT	*-0.55*	FAM46C	plasma cells, others	*-0.56*	ABCC3	Injury-induced	*-0.52*	SLC12A6	normal kidney	*-0.59*
COL4A1	IRITD3	*-0.81*	LSP1	plasma cells, others	*-0.55*	CYP3A4	kidney (PCT)	*-0.55*	RBBP8	Mitosis-related	*-0.52*	ACAT1	normal kidney	*-0.58*
FHL2	Injury-induced	*-0.81*	IGLV1-40	IGT	*-0.54*	MARC2	normal kidney	*-0.53*	RASD1	IRITD3	*-0.50*	HYAL1	normal kidney	*-0.58*
THBS2	IRITD5	*-0.80*	RING1	plasma cells	*-0.53*	GSTA1	normal kidney	*-0.53*	KRT18	IRITD3	*-0.50*	SUSD2	normal kidney	*-0.58*
PXDN	Injury-induced	*-0.79*	CD79A	plasma cells	*-0.53*	KIAA1147	normal kidney	*-0.53*	SERPINA3	Injury-induced	*-0.48*	KMO	normal kidney	*-0.57*
VCAN	IRRAT	*-0.79*	IGKC	IGT	*-0.53*	CTXN3	normal kidney	*-0.53*	TRNP1	injury-induced	*-0.48*	PRLR	normal kidney	*-0.57*
AFAP1	injury-induced	*-0.78*	IGHG1	IGT	*-0.52*	SERPINA6	normal kidney	*-0.53*	C2CD4A	Injury-induced	*-0.48*	DDC	normal kidney	*-0.57*
ESYT1	Injury-induced	*-0.77*	IGK	IGT	*-0.52*	ZNF540	normal kidney	*-0.52*	TTC39A	injury-induced	*-0.47*	SHBG	normal kidney	*-0.57*
TPM1	Injury-induced	*-0.77*	CELF2	plasma cells, others	*-0.51*	MASP1	normal kidney	*-0.52*	LAPTM4B	Injury-induced	*-0.47*	MSRA	normal kidney	*-0.57*
MTHFD1L	Injury-induced	*-0.77*	IGLJ3	IGT	*-0.51*	ST7	normal kidney	*-0.52*	SEMA4B	Injury-induced	*-0.46*	SUCLG1	normal kidney	*-0.56*

[A] All *P* values were significant at <0.0001.; (parenchyma) indicate significant expression in normal kidney: injury-induced indicates significantly increased in kidneys with clinically-defined AKI.

Abbreviations: PC, principal component; PBT, pathogenesis-based transcript set; SCC, Spearman correlation coefficient; IRRAT, injury/repair associated transcripts (human kidney); IRITD3, tissue injury and repair associated transcripts; IRITD5, tissue injury and repair associated transcripts; MCAT, Mast cell-associated transcripts; QCMAT, constitutive macrophage transcripts; AKI, acute kidney injury.

Supplemental Table 7. Injury features of the injury archetype groups in 2479 biopsies with No rejection: 459 early, 996 late, and all 2479

Archetype groups		Time of biopsy post-transplant (days)		Mean±SEM scores												
				eGFR[A]	Macrophage-associated transcript sets		Recent (acute) injury-induced transcript sets		Atrophy-fibrosis – associated features				Normal kidney transcripts	Injury PC scores		
Group	N	Median	Mean±SEM[A]		AMAT1[A]	QCMAT[A]	IRITD3[AB]	IRRAT[AB]	IGT[AB]	MCAT[AB]	ci>1$_{Prob}$ classifier[AB]	Histologic ci-lesion[A]	KT1[A]	PC1[A]	PC2[A]	PC3[A]
Early (N=459) ≤42 days[C]																
**Normal**	136	20	**22±0.88 a**	**45.5±2.56 a**	*0.45±0.03 a*	0.39±0.03 a	*0.05±0.01 a*	*0.18±0.03 a*	-0.07±0.04 a	*-0.22±0.05 a*	*0.09±0.01 a*	*0.49±0.07 a*	**-0.02±0.02 a**	*-2.4±0.09 a*	**-0.9±0.06 a**	*-0.28±0.06 a*
**AKI1**	184	20	**22±0.83 a**	21.9±2.23 b	0.51±0.02 a	*0.37±0.02 a*	0.18±0.01 b	0.76±0.03 b	*-0.24±0.03 b*	0.06±0.05 b	0.15±0.01 b	0.69±0.09 b	-0.13±0.01 b	-0.5±0.09 b	-1.7±0.07 b	**1.08±0.04 b**
**AKI2**	121	14	*16±0.88 b*	*20.9±2.08 b*	**1.08±0.03 b**	**0.92±0.04 b**	**0.37±0.01 c**	**1.37±0.03 c**	**-0.01±0.06 a**	**0.10±0.07 b**	**0.20±0.01 c**	**0.73±0.09 b**	*-0.39±0.02 c*	**1.42±0.11 c**	*-2.9±0.08 c*	-0.02±0.06 c
Late (N=996) >365 days																
**Normal**	361	1160	1922±100 a	**55.2±1.6 a**	*0.22±0.01 a*	*0.19±0.01 a*	*-0.03±0.01 a*	*-0.24±0.02 a*	0.56±0.04 a	*0.96±0.04 a*	*0.23±0.01 a*	*0.98±0.06 a*	**0.03±0.01**	*-2.57±0.06 a*	0.67±0.03 a	-0.16±0.03 a
**AKI1**	87	856	1632±193 a	40.6±3.8 b	0.34±0.02 b	0.27±0.02 b	0.07±0.01 b	0.25±0.04 b	*0.35±0.07 b*	1.19±0.08 b	0.32±0.02 b	1.52±0.17 b	-0.01±0.01 b	-1.09±0.13 b	0.24±0.07 b	**0.95±0.04 b**
**AKI2**	13	760	*812±97 a*	36.8±9.2 b	0.77±0.09 c	**0.70±0.1 c**	**0.36±0.04 c**	**1.17±0.11 c**	1.39±0.2 c	1.04±0.19 b	0.36±0.04 b	1.50±0.65 c	-0.4±0.12 c	1.90±0.36 c	*-1.34±0.12 c*	*-0.89±0.29 c*
**Mild CKD**	249	2086	2577±123 b	52.5±1.8 a	0.56±0.02 d	0.43±0.01 d	0.07±0.01 b	0.23±0.02 b	**2.03±0.05 d**	2.08±0.04 c	0.45±0.01 c	1.36±0.07 b	-0.06±0.01 d	-0.33±0.08 d	**1.50±0.05 d**	-0.17±0.04 a
**CKDAKI**	286	2434	**3120±148 c**	*34.9±1.3 b*	**0.78±0.02 c**	0.55±0.01 e	0.26±0.01 d	0.89±0.02 d	1.69±0.06 c	**2.68±0.05 d**	**0.79±0.01 d**	**2.08±0.07 c**	*-0.25±0.01 e*	**2.62±0.09 e**	1.23±0.05 e	0.50±0.04 d
All (N=2479)																
**Normal**	920	239	867±37 a	**52.1±1.5 a**	*0.26±0.01 a*	*0.24±0.01 a*	*-0.0009±0.005 a*	*-0.10±0.02 a*	0.27±0.02 a	0.52±0.04 a	0.17±0.01 a	0.76±0.06 a	**0.01±0.01 a**	*-2.57±0.05 a*	0.06±0.05 a	-0.16±0.02 a
**AKI1**	593	82	325±17 b	29.7±2.1 b	0.44±0.03 b	0.33±0.03 b	0.14±0.01 b	0.55±0.03 b	-0.0±0.07 b	0.60±0.06 b	0.23±0.01 b	0.96±0.08 b	-0.08±0.02 b	-0.72±0.09 b	-0.9±0.07 b	**0.99±0.06 b**
**AKI2**	190	22	*95±128 b*	*25.5±1.2 b*	**1.00±0.02 c**	**0.86±0.01 c**	**0.36±0.01 c**	**1.32±0.02 c**	0.28±0.05 a	0.26±0.04 c	0.23±0.01 b	0.86±0.06 b	-0.3±0.01 c	1.42±0.07 c	*-2.54±0.05 c*	-0.16±0.04 a
**Mild CKD**	355	1316	1958±107 c	51.5±1.6 a	0.59±0.01 d	0.45±0.01 d	0.08±0.005 d	0.26±0.02 d	**1.96±0.04 c**	1.96±0.04 d	**0.42±0.01 c**	1.29±0.06 c	-0.07±0.01 b	-0.38±0.07 d	**1.28±0.04 d**	*-0.23±0.04 a*
**CKDAKI**	421	1430	**2356±51 d**	34.6±1.1 c	0.79±0.01 e	0.56±0.01 e	0.28±0.004 e	0.96±0.01 e	1.46±0.02 d	**2.46±0.03 e**	0.75±0.004 d	**2.01±0.03 d**	*-0.28±0.01 d*	**2.58±0.04 e**	0.87±0.03 e	0.60±0.02 c

[A] Compact lettering display: Means not sharing any letter are significantly different by all pairwise comparisons at the 5% level of significance.
[B] These variables were used in the injury AA.
[C] Six biopsies called CKD and 12 called CKDAKI archetypes are not shown because there were too few to analyze.
Bold underlined scores are the highest per column in each biopsy group; Italics signifies lowest in column.

Supplemental Table 8. Spearman correlations on -New PBT scores with archetype and PC scores in all 4502 biopsies.

	**Spearman Correlation Coefficient (ρ) with principal component scores**[A]			**Spearman Correlation Coefficient (ρ) with injury archetype scores**[B]				
**Gene set**	**Injury PC1 score**	**Injury PC2 score**	**Injury PC3 score**	**Mild CKD score**	**CKDAKI score**	**AKI1 score**	**AKI2 score**	**Normal score**
PT_New1 oxidative stress	**-0.64**	0.002	0.10	0.06	**-0.50**	0.30	-0.22	**0.48**
tL_New1 oxidative stress	**-0.67**	0.03	0.16	0.09	**-0.53**	0.37	-0.28	**0.45**
DCT_New1 oxidative stress	**-0.64**	0.03	0.22	0.06	**-0.47**	0.40	-0.29	**0.43**
PT_New2 hypoxia	**0.70**	-0.23	0.38	-0.32	**0.53**	0.19	0.29	**-0.70**
TAL_New2 hypoxia	**0.30**	-0.23	0.32	-0.28	**0.23**	0.22	0.18	**-0.34**
DCT_New2 hypoxia	**0.43**	-0.14	0.32	-0.18	**0.30**	0.22	0.14	**-0.51**
PT_New3 interferon response	-0.15	-0.15	**0.31**	**-0.18**	-0.07	**0.34**	-0.001	0.07
TAL_New3 interferon response	**0.63**	-0.29	0.23	-0.33	**0.46**	0.07	0.38	**-0.53**
DCT_New3 interferon response	**0.69**	-0.28	0.25	-0.38	**0.54**	0.04	0.39	**-0.55**
PT_New4 EMT	**0.89**	-0.20	0.16	-0.31	**0.69**	-0.08	0.40	**-0.74**
TAL_New4 EMT	**0.89**	-0.19	0.21	-0.33	**0.72**	-0.06	0.37	**-0.73**
DCT_New4 EMT	**0.78**	-0.25	0.31	-0.38	**0.62**	0.08	0.35	**-0.69**

[A] Strongest association per row is bolded.
[B] Highest and lowest correlations per row are bolded.
Abbreviations: PC, principal component; EMT, epithelial mesenchymal transition; DCT, distal convoluted tubule; New1, oxidative stress; New2, hypoxia; New3 interferon response; New4, mesenchymal transition; PT, proximal tubule; TAL, thick ascending limb; tL, thin limb

Supplemental Table 9. Associations of various gene sets with risk of 3-year graft loss in No rejection biopsies (N=2479) (arranged by *P* value) and correlation with eGFR and donor age.

	**3-year graft loss in NR2479, arranged by *P* value**		**Correlations with eGFR**		**Correlations with donor age**	
**Variable**	**Hazard Ratio**	***P value***	**Spearman Correlation Coefficient (ρ)**	***P value***	**Spearman Correlation Coefficient (ρ)**	***P value***
PT_New4 EMT	2.04	*2E-08*	-0.56	*2E-86*	0.19	*1E-10*
DCT_New4 EMT	1.87	*3E-08*	-0.56	*1E-89*	0.17	*7E-09*
TAL_New4 EMT	1.86	*8E-08*	-0.56	*3E-87*	0.19	*1E-10*
PT_New2 hypoxia	1.84	*2E-07*	-0.54	*3E-80*	0.16	*4E-08*
IRRAT30	1.72	*3E-06*	-0.56	*2E-89*	0.19	*9E-11*
IRITD3	1.53	*2E-04*	-0.52	*3E-73*	0.16	*4E-08*
DCT_New3 interferon response	2.03	*9E-07*	-0.52	*2E-74*	0.15	*1E-06*
TAL_New3 interferon response	1.87	*3E-06*	-0.50	*2E-68*	0.12	*6E-05*
IRITD5	1.29	*0.02*	-0.35	*3E-32*	0.12	*4E-05*
DCT_New2 hypoxia	1.34	*0.008*	-0.36	*2E-33*	0.11	*4E-04*
PT_New1 oxidative stress	0.96	*0.71*	0.16	*2E-07*	-0.03	*3E-01*
tL_New1oxidative stress	1.01	*0.95*	0.19	*8E-10*	-0.05	*8E-02*
TAL_New2 hypoxia	1.14	*0.23*	-0.31	*1E-25*	0.12	*4E-05*
DCT_New1 oxidative stress	1.04	*0.73*	0.08	*0.006*	0.06	*0.04*
PT_New3 interferon response	1.20	*0.16*	-0.17	*2E-08*	-0.01	*7E-01*
Injury PC1	1.89	*1E-08*	-0.47	*1E-59*	0.15	*4E-07*
Injury PC2	1.01	*0.93*	0.32	*2E-26*	-0.10	*6E-04*
Injury PC3	1.89	*7E-07*	-0.44	*3E-50*	0.19	*8E-11*

Abbreviations: DCT, distal convoluted tubule; New1, oxidative stress; New2, hypoxia; New3, interferon response; New4, mesenchymal transition; PT, proximal tubule; TAL, thick ascending limb; tL, thin limb; IRRAT30, injury/repair associated transcripts (human kidney) N=30; IRITD3, tissue injury and repair associated transcripts, day 3; IRITD5, tissue injury and repair associated transcripts, day 5; PC1/2/3, principal components 1/2/3;

Supplemental Table 10. Correlations of 13 selected genes from injury genes from single nuclei cell injury states in 4502 No rejection biopsies ordered by highest correlation with injury PC1 (14)

Gene Symbol	Gene and interpretation	Annotation in new PT/tL/TAL/DCT injury states in AKI	Spearman Correlation Coefficient (ρ) with Injury PC1	Spearman Correlation Coefficient (ρ) with Injury PC2	Spearman Correlation Coefficient (ρ) with Injury PC3
IFITM3	interferon induced transmembrane protein 3	PT_New4, TAL_New3, DCT_New3	0.72	-0.19	0.11
VCAM1	vascular cell adhesion molecule 1 EMT (PT-New 4) marker gene	PT_New4	0.70	-0.14	0.09
HIF1A	hypoxia inducible factor 1, alpha subunit (basic helix-loop-helix transcription factor)	DCT_New4	0.70	-0.34	0.14
MET	MET proto-oncogene, receptor tyrosine kinase (EMT marker gene)	PT_New2, TAL_New4, DCT_New4	0.60	-0.15	0.35
JUN	jun proto-oncogene enriched transcription Factor New 4	PT_New4, TAL_New4, DCT_New3	0.51	-0.29	-0.08
IGFBP7	insulin like growth factor binding protein 7 (upregulated in PT and TALs)	PT_New4, TAL_New3	0.31	-0.03	0.13
SPP1	secreted phosphoprotein 1, encoding Osteopontin (upregulated in virtually all non-leukocyte kidney cell types)	PT_New4, TAL_New3, DCT_New3,	0.28	-0.43	0.30
SLC2A1	solute carrier family 2 (facilitated glucose transporter), member 1 canonical TAL marker gene; hypoxia signature;	TAL_New2, DCT_New2	0.24	-0.10	0.15
SERPINA1	serpin peptidase inhibitor, clade A (alpha-1 antiproteinase, antitrypsin), member 1(inflammation response, marker gene for PT-New 3)	PT_New3, TAL_New3,	0.18	-0.17	-0.12
MYO5B	myosin VB (hypoxia-associated gene; marker gene for PT-New 2)	PT_New2, TAL_New4	-0.14	-0.14	0.31
NQO1	NAD(P)H dehydrogenase, quinone 1 (Oxidative stress-related gene; marker gene for PT-New 1)	PT_New1	-0.14	-0.13	0.27
LRP2	LDL receptor related protein 2	DCT_New1	-0.45	0.09	0.33
ALDOB	aldolase B, fructose-bisphosphate (oxidative stress marker gene):	PT_New1, tL_New1, DCT_New1	-0.69	0.23	0.13

**Supplemental Table 11.** Mean gene expression in injury groups of top genes correlating positively with injury PC3 in 2479 No rejection biopsies.							
**Gene Symbol**	**Description**	**Spearman correlation coefficient (ρ)** [A]	**Expression in injury archetype group**				
			**Normal**	**AKI1**	**AKI2**	**Mild CKD**	**CKDAKI**
PLEKHH1	pleckstrin homology domain containing, family H (with MyTH4 domain) member 1	0.61	*187*	**264**	209	199	**257**
KLF8	Kruppel-like factor 8	0.58	*60*	**89**	77	72	**89**
PARD3	par-3 family cell polarity regulator	0.58	*254*	**338**	313	262	**316**
PTPRK	protein tyrosine phosphatase, receptor type, K	0.57	*884*	**1078**	981	947	**1117**
ZDHHC23	zinc finger, DHHC-type containing 23	0.57	201	**262**	**225**	*197*	215
USP43	ubiquitin specific peptidase 43	0.57	*53*	**74**	68	54	**70**
NBEA	neurobeachin	0.56	*50*	**70**	58	54	**66**
SAMD12	sterile alpha motif domain containing 12	0.56	324	**385**	*320*	336	**406**
TUFT1	tuftelin 1	0.56	398	**475**	**433**	*396*	**433**
SEMA6A	sema domain, transmembrane domain (TM), and cytoplasmic domain, (semaphorin) 6A	0.56	*173*	**235**	188	191	**204**
MYRF	myelin regulatory factor	0.56	*159*	**211**	198	180	**255**
PPP1R9A	protein phosphatase 1, regulatory subunit 9A	0.55	365	**435**	*347*	367	**426**
MYEF2	myelin expression factor 2	0.55	164	**198**	168	*161*	**186**
SPRY4	sprouty RTK signaling antagonist 4	0.55	*198*	**261**	248	212	**245**
NF2	neurofibromin 2 (merlin)	0.55	*592*	**733**	**751**	619	697
MTMR2	myotubularin related protein 2	0.54	*172*	**216**	185	173	**179**
FAT1	FAT atypical cadherin 1	0.54	*1947*	**2465**	2370	1982	**2431**
SMARCA1	SWI/SNF related, matrix associated, actin dependent regulator of chromatin, subfamily a, member 1	0.54	*231*	**321**	292	263	**323**
KIAA0556	KIAA0556	0.54	*168*	**211**	199	174	**205**
XPO5	exportin 5	0.54	*208*	**250**	228	221	**233**

The highest two in each row are bolded; the lowest is italics.

[A] All *P* values were < 0.0001

**Supplemental Table 12.** Top 10 genes in all 4502 biopsies by Spearman correlation coefficient correlating positively and negatively with Injury archetype scores[A]														
**Normal**			**AKI1**			**AKI2**			**Mild CKD**			**CKDAKI**		
**Gene symbol**	**PBT annotation or cell expression**	**SCC (ρ)**	**Gene symbol**	**PBT annotation or cell expression**	**SCC (ρ)**	**Gene symbol**	**PBT annotation or cell expression**	**SCC (ρ)**	**Gene symbol**	**PBT annotation or cell expression**	**SCC (ρ)**	**Gene symbol**	**PBT annotation or cell expression**	**SCC (ρ)**
Positive correlation														
HINT2	normal kidney	0.67	SPTBN1	injury-induced	0.55	SLC16A3	injury-induced	0.61	JCHAIN	Plasma cells	0.62	LAMC2	epithelial, endothelial	0.76
GLYCTK	normal kidney	0.67	UNC13B	normal kidney	0.55	SCARB2	IRRAT950	0.60	IGKV1-39	IGT	0.62	CXCL6	epithelial, endothelial	0.75
CLCN5	normal kidney	0.67	PTPN3	normal kidney	0.55	CEBPB	IRITD3	0.60	IGKV1-27	IGT	0.62	MMP7	IRITD5	0.71
GLTPD2	normal kidney	0.67	MTMR2	injury-induced	0.54	S100A9	QCMAT	0.59	IGHG1	IGT	0.62	PROM1	atrophy-fibrosis/AKI	0.70
TRPM3	normal kidney	0.67	KIF21A	normal kidney	0.53	PTX3	IRRAT	0.59	IGHG3	IGT	0.62	LAMA3	atrophy-fibrosis/AKI	0.70
PDP2	normal kidney	0.66	BEND7	normal kidney	0.52	CLIC1	IRITD3	0.59	IGKV1-5	IGT	0.61	CPA3	MCAT	0.70
THEM6	normal kidney	0.66	KIF3B	normal kidney	0.52	ARPC1B	IRITD3	0.58	IGKC	IGT	0.61	TPSAB1	Mast cells	0.69
GGT6	normal kidney	0.66	ARHGEF12	normal kidney	0.51	UBE2S	Injury-induced	0.58	IGK	IGT	0.60	ANXA3	atrophy-fibrosis/AKI	0.69
AMT	normal kidney	0.66	PARD3	normal kidney	0.51	TOP2A	injury-induced	0.57	FAM46C	plasma cells	0.60	CLIC6	atrophy-fibrosis/AKI	0.69
NAGS	normal kidney	0.65	KIAA0895	normal kidney	0.51	S100A8	IRRAT	0.57	IGLL5	IGT	0.57	CXCL1	atrophy-fibrosis/AKI	0.69
Negative correlation														
FHL2	Injury-induced	-0.79	CD79A	Plasma cells	-0.59	ZNF540	normal kidney	-0.58	ABCC3	injury-induced	-0.52	SLC12A6	normal kidney	-0.59
TIMP1	injury-induced	-0.77	IGLL3P	IGT	-0.59	KIAA1147	normal kidney	-0.56	RASD1	IRITD3	-0.51	KMO	normal kidney	-0.58
COL4A1	IRITD3	-0.77	IGKC	IGT	-0.57	ESR1	normal kidney	-0.56	RBBP8	mitosis	-0.50	SUSD2	normal kidney	-0.58
VCAN	IRRAT	-0.77	IGK	IGT	-0.57	CYP3A4	Kidney (PCT)	-0.55	LAPTM4B	injury-induced	-0.49	SUCLG1	normal kidney	-0.57
FHL2	Injury-induced	-0.76	CORO1A	T,NK,mac	-0.57	CYP4X1	normal kidney	-0.55	KRT18	IRITD3	-0.47	DDC	normal kidney	-0.57
THBS2	IRITD5	-0.76	LSP1	plasma cells	-0.56	CTXN3	normal kidney	-0.54	TRNP1	injury-induced	-0.47	MTTP	normal kidney	-0.57
COL4A2	IRITD3	-0.76	IGLV1-40	IGT	-0.56	ST7	normal kidney	-0.54	RAB13	injury-induced	-0.47	PRLR	normal kidney	-0.57
PXDN	Injury-induced	-0.75	IGHG1	IGT	-0.56	ROBO2	podocytes	-0.54	OLFM4	IRRAT	-0.47	MSRA	normal kidney	-0.57
ANXA2P2	IRITD3	-0.74	ZBP1	plasma cells	-0.56	PMPCB	normal kidney	-0.54	TRNP1	Injury-induced	-0.46	PRLR	normal kidney	-0.57
AXL	IRITD5	-0.74	CELF2	plasma cells	-0.56	SERPINA6	normal kidney	-0.54	WFDC2	IRITD3	-0.46	MTTP	normal kidney	-0.56
Abbreviations: IRITD3, Injury-repair induced transcripts peaking day 3; IRITD5, Injury-repair induced transcripts peaking day 5; IRRAT, Injury/repair associated transcripts; IGT, immunoglobulin transcripts; MCAT, Mast cell-associated transcripts; QCMAT, constitutive macrophage transcripts; PBT, pathogenesis-based transcript set; SCC, Spearman correlation coefficient; AKI, acute kidney injury; CKD, chronic kidney disease;														

Supplemental Table 13. Biological processes correlating with each injury PC and archetype score in 2479 No rejection biopsies.

**Injury PC1/2/3 scores (e.g. typical gene)<br>Includes "summary name"**			
**Correlation**	**Injury PC1<br>"Normal vs. Injury/failed repair"**	**Injury PC2<br>"early AKI vs late CKD"**	**Injury PC3<br>"Inflammation vs Epithelial remodeling"**
**Positive**	AKI-induced (including failed repair)<br>e.g. VIM, VCAN, ANXA1	Late CKD-associated (plasma cells, mast cells)<br>e.g. IGKC, FCER1A, CPA3	Epithelial cell structure, development<br>e.g. PARD3, PTPRK
**Negative**	Metabolism, transport<br>e.g. SLC12A6, TRPM3, HINT2, MRSA	Early AKI and mitosis<br>e.g. OLFM4, RAB20	Inflammation, T/NK cells<br>e.g. LSP1, ZAP70, PRF1

**Injury archetype scores (e.g. typical gene)**					
	**Normal**	**AKI1**	**AKI2**	**Mild CKD**	**CKDAKI**
**Positive**	Metabolism, transport<br>e.g. TRPM3, HINT2	Epithelial cell structure, development<br>e.g. PARD3	Recent injury, failed repair<br>e.g. CLIC1, SERPINA3	Plasma cells<br>e.g. IGHG1, IGKC	Atrophy-fibrosis (e.g. CXCL6), mast cells (e.g. CPA3), recent injury (ANXA1)
**Negative**	Recent injury, failed repair<br>e.g. TIMP1, VCAN	Inflammation especially plasma cells<br>e.g. LSP1, CD79A, IGHG1	Various functions<br>e.g. CYP3A4, MARC2	Recent injury or failed repair<br>e.g. RASD1, KRT18	Metabolism, transport<br>e.g. SLC12A6, MSRA

Details are listed in Supplemental Table 4 and Supplemental Table 6.

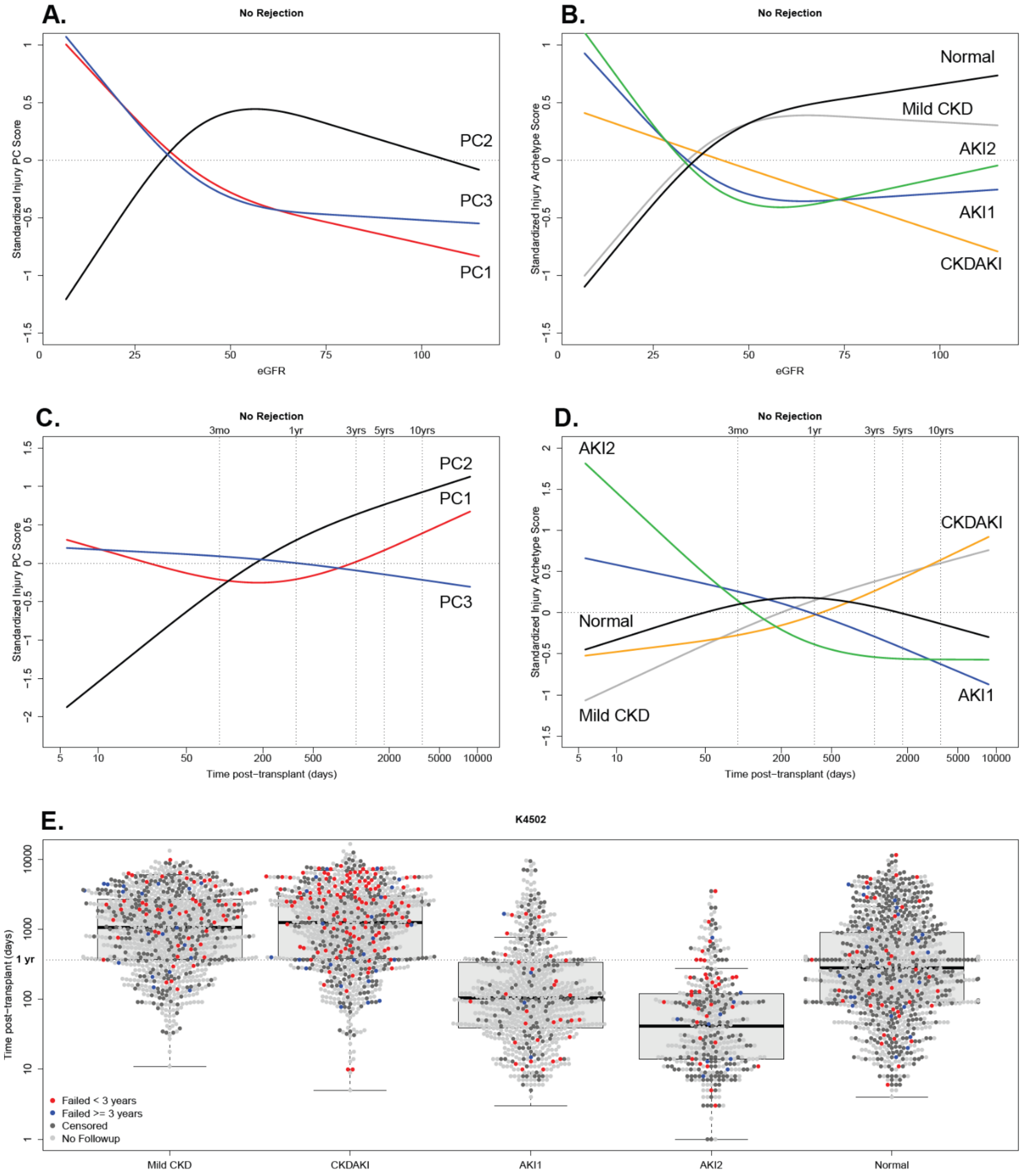

Supplemental Figure 1. Relationships in 2479 No rejection biopsies between Injury principal component scores, Injury archetypes, eGFR, and time post-transplant. Restricted cubic splines showing the relationship between Injury PC1, PC2, PC3 and eGFR in **A)** Restricted cubic splines showing the relationship between Injury archetypes and eGFR in **B)** Restricted cubic splines showing the relationship **C)** Injury archetypes and time post-transplant and between Injury PC1, PC2, PC3 and time post-transplant. Scores were standardized before analysis so they could be shown on the same scale. **E)** Beeswarm/boxplots showing time post-transplant vs. Injury archetype groups in N=4502 samples. Dots represent biopsies and are colored by transplant status. Abbreviations: PC1/2/3 – principal components 1/2/3; eGFR – estimated glomerular filtration rate

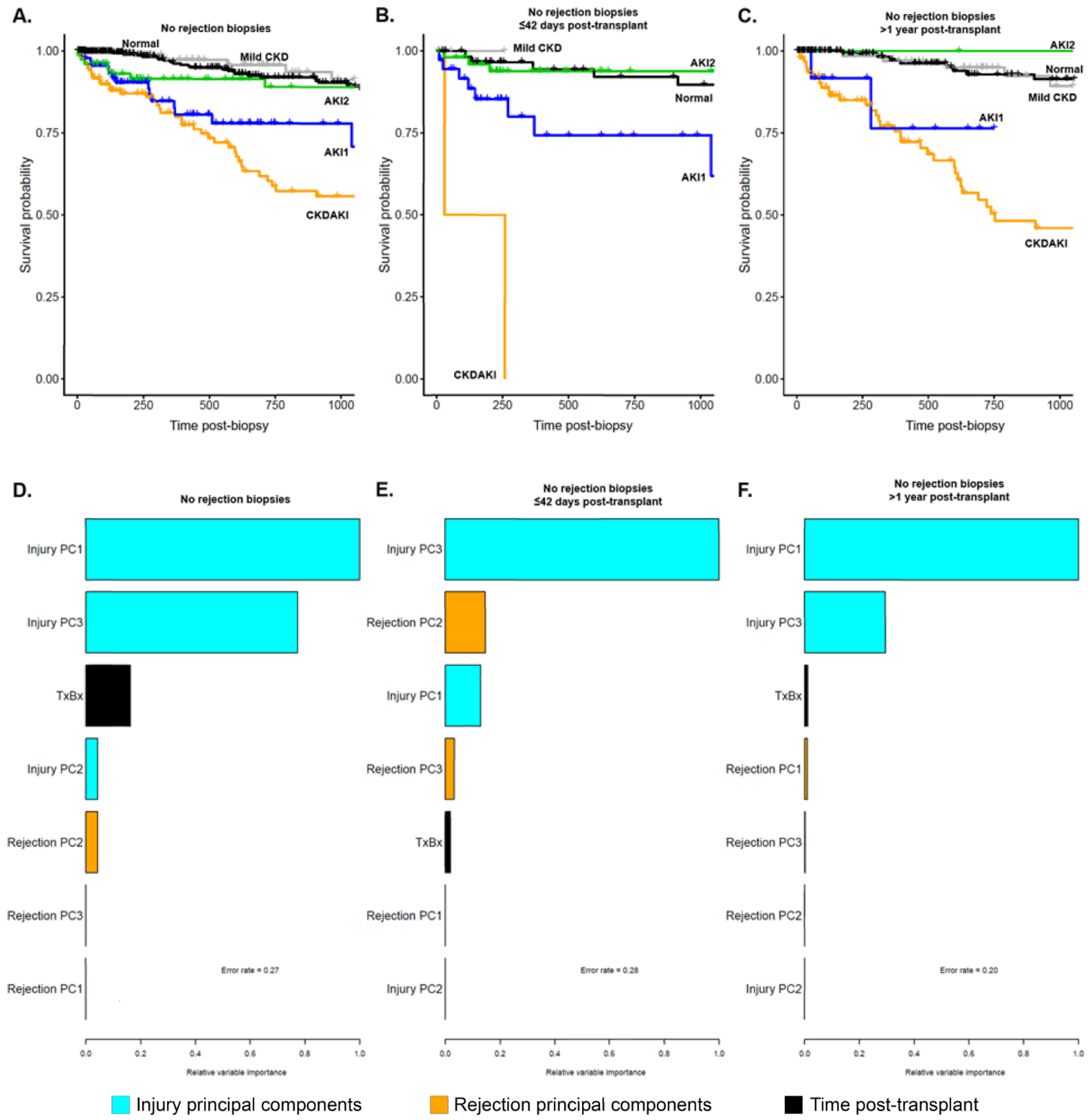

Supplemental Figure 2. Relationship between injury and 3-year post-biopsy death-censored survival (one random biopsy per kidney) in No rejection biopsies. **A)** Kaplan-Meier plots for the injury archetype groups in No rejection biopsies. **B)** Kaplan-Meier plots for the injury archetype groups in No rejection biopsies ≤42 days. **C)** Kaplan-Meier plots for the injury archetype groups in No rejection biopsies >1 year. Panels D-F show relative variable importance plots from random survival forest analyses using injury PC1, PC2, and PC3, rejection PC1, PC2, and PC3, and time of biopsy post-transplant (TxBx) as predictors. **D)** in all No rejection biopsies, **E)** in No rejection biopsies ≤42 days post-transplant and **F)** in No rejection biopsies >1 year post-transplant.

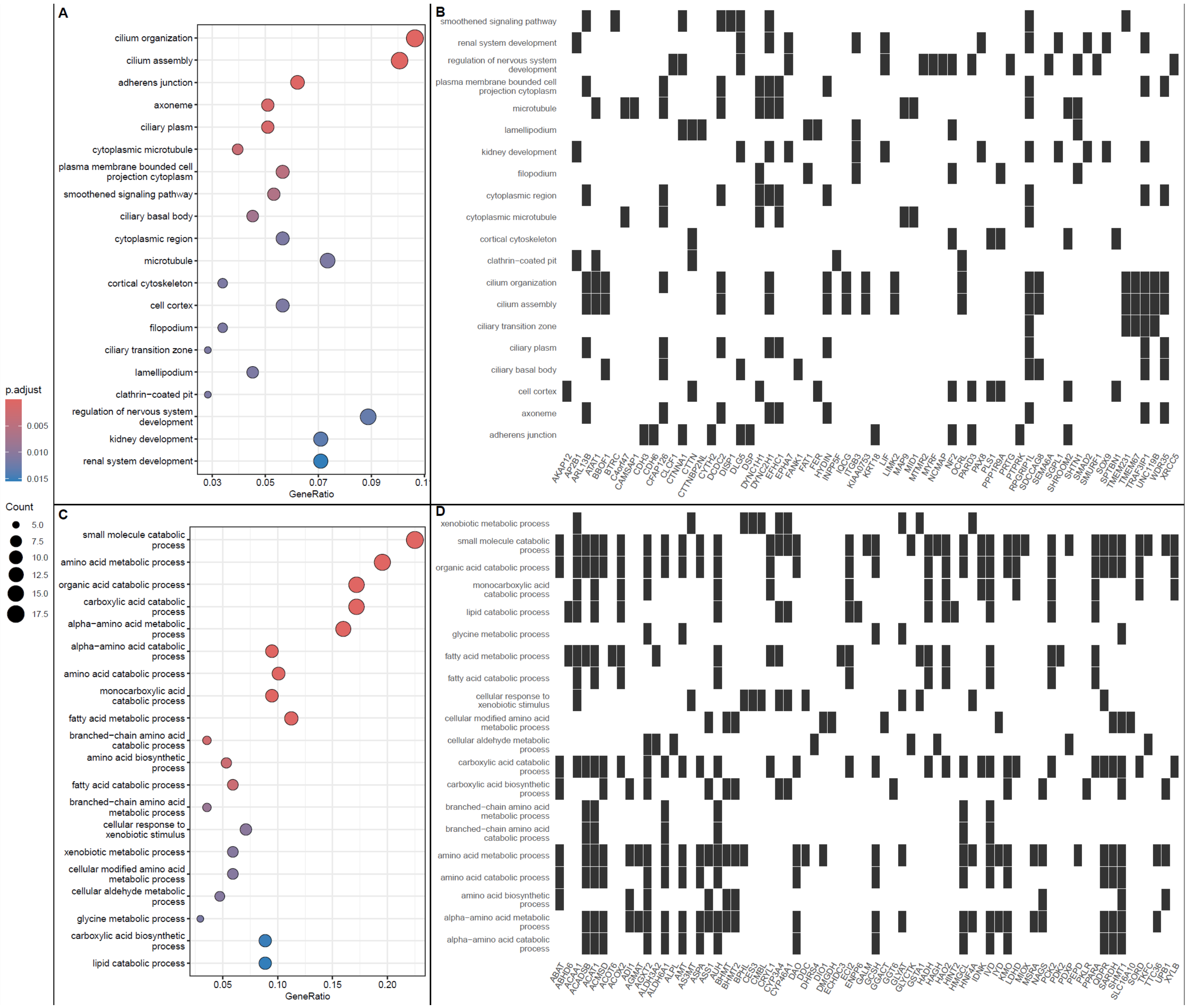

Supplemental Figure 3. Visualizing the GO categories. **A**) Top 20 GO categories overrepresented by the top 200 genes correlating positively with Injury PC3, y-axis ordered by increasing adjusted *P* value. Gene ratio denotes the number of differentially expressed genes found in our analyses vs the number of genes associated with each term overall. **B)** The same top GO categories as in “A”, showing the distribution of all top 200 genes that were found in any of those 20 categories. **C** and **D**) Same as “A” and “B” but using the top 200 genes correlating negatively with Injury PC1.

Supplemental Results

Expression of the PC3-correlated genes in each archetype group (**Supplemental Table 11**). Unlike the usual AKI transcripts, most of the top transcripts correlated with PC3 genes were increased in all injury groups compared to Normal biopsies but expressed more strongly in AKI1 vs. AKI2 and CKDAKI vs. Mild CKD. In our cell panel (see Methods), the PC3-correlated genes were expressed in epithelial cells (RPTEC) rather than inflammatory cells.

Gene Ontology (GO) analyses. From the above analysis, injury can increase or decrease the expression of genes expressed in normal kidneys. **Supplemental Table 5** compares GO terms for two sets of genes expressed in normal kidneys: the top PC3-correlated genes and the top PC1-anticorrelated genes. The top PC3-correlated genes were expressed in normal kidneys but increased by injury. The top PC1-anticorrelated genes were also expressed in normal kidneys but decreased by AKI, reflecting the dedifferentiation associated with AKI.

Supplemental Figure 3, A and **B** show further details of the GO terms overrepresented by the top 200 genes correlating positively with PC3 in No rejection biopsies. The GO terms for PC3 genes were associated with epithelial organization, cytoskeleton, development, and tight junctions, but not metabolism. In contrast, the GO categories overrepresented by the top PC1 anti-correlated genes were all related to metabolism or mitochondrial function (**Supplemental Figure 3, C** and **D**).

Features of the five injury groups in No rejection biopsies. **Supplemental Table 7** shows TxBx, eGFR, molecular injury scores, and histologic fibrosis (ci) scores, for the 5 injury archetype groups in No rejection biopsies. Compared to AKI2, AKI1 had macrophage transcripts and less inflammation.

Early biopsies included 136 Normal, 184 AKI1, and 121 AKI2; there were too few with Mild CKD or CKDAKI for reliable estimates (**Supplemental Table 7**). Despite similar TxBx and depression of eGFRs, AKI1 had less of the typical constellation of AKI changes i.e. less AKI and macrophage transcripts and less dedifferentiation (loss of normal kidney transcripts, KT1). Early biopsies had minimal CKD changes.

Late biopsies (>1 year) included Normal (361), Mild CKD (249), or CKDAKI (286), plus some AKI1 biopsies (87) but few AKI2 biopsies (13). Normal biopsies had the lowest scores for all AKI and CKD features and the highest eGFR. Mild CKD and CKDAKI had elevated histologic fibrosis scores, fibrosis classifier scores, and IGT (1) compared to Normal. Compared to Mild CKD, CKDAKI had higher AKI-induced scores and lower eGFR. CKDAKI had more AKI, mast cell (2), and macrophage transcripts than mild AKD, whereas Mild CKD had more immunoglobulin transcripts.

The features of the whole 2479 No rejection biopsy population reflected the above trends (**Supplemental Table 7**), as did the whole 4502 population (**Table 6**).

Top genes correlating with injury PC1, PC2, and PC3 scores in No rejection biopsies (Supplemental Table 4). The top genes correlating positively with injury PC1 were almost all typical AKI-induced genes previously annotated in human kidney transplants (IRRAT) (3) or mouse kidney transplant models (IRITD3) (4) e.g. VIM, VCAN, NNMT, ANXA1, and ANXA2. The top genes correlating negatively with PC1 (i.e. with decreased expression) were expressed in Normal biopsies (e.g. solute carrier SLC12A6 and dehydrogenase IVD) and related to solute transport and metabolism.

The top positive PC2-correlated genes (i.e. associated with CKD/atrophy-fibrosis) were related to mast cells (e.g. FCER1A, MS4A2) or were immunoglobulin transcripts (IGT). The top negative PC2 genes were induced in AKI (e.g. OLFM4 and RAS oncogene member RAB20).

The top positive PC3 correlated genes were expressed in normal kidneys but increased by injury (e.g. PARD3). The genes negatively correlating with injury PC3 were related to inflammatory cells e.g. LSP1, ZAP70.

These correlations with PC scores in all 4502 biopsies (**Table 3**) were similar to those in the No rejection biopsies.

Top genes correlating with injury archetype scores (Supplemental Table 6). The genes most positively correlated with the Normal archetype score were highly expressed in normal kidneys (e.g. TRPM3) while the top negative genes were AKI-induced (e.g. TIMP1) i.e. the reverse of PC1.

The top genes positively correlated with AKI1 were expressed in Normal kidneys but increased in AKI1 (e.g. PARD3). Genes correlated negatively with AKI1 were related to plasma cells e.g. IGTs and CD79A.

The top genes positively correlated with the AKI2 score were AKI-induced (e.g. SERPINA3), while the top negatives were expressed in normal kidney (e.g. CYP3A4).

The top genes positively correlated with the Mild CKD archetype score were all expressed in plasma cells e.g. IGHG1, while the negatives were AKI-induced e.g. SERPINA3 and OLFM4.

The top genes positively correlated with the CKDAKI score were a combination of recent injury-induced genes (e.g. MMP7, LAMC2) and atrophy-fibrosis-related genes e.g. CXCL6 and mast cell gene CPA3, both highly correlated with atrophy-fibrosis (5). The negatively correlated genes were highly expressed in Normal kidneys e.g. SLC12A6.

These findings were similar in all 4502 biopsies (**Supplemental Table 12**).

Reference List

1. Einecke G, et al. Expression of B cell and immunoglobulin transcripts is a feature of inflammation in late allografts. *Am J Transplant.* 2008;8(7):1434-43.
2. Mengel M, et al. Molecular correlates of scarring in kidney transplants: the emergence of mast cell transcripts. *Am J Transplant.* 2009;9(1):169-78.
3. Famulski KS, et al. Molecular phenotypes of acute kidney injury in kidney transplants. *J Am Soc Nephrol.* 2012;23(5):948-58.
4. Famulski KS, et al. Transcriptome analysis reveals heterogeneity in the injury response of kidney transplants. *Am J Transplant.* 2007;7(11):2483-95.
5. Venner JM, et al. Relationships among injury, fibrosis, and time in human kidney transplants. *Jci Insight.* 2016;1(1):e85323.
